# Hantavirus: an overview and advancements in therapeutic approaches for infection

**DOI:** 10.3389/fmicb.2023.1233433

**Published:** 2023-10-12

**Authors:** Samia Afzal, Liaqat Ali, Anum Batool, Momina Afzal, Nida Kanwal, Muhammad Hassan, Muhammad Safdar, Atif Ahmad, Jing Yang

**Affiliations:** ^1^CEMB, University of the Punjab, Lahore, Pakistan; ^2^Department of Biological Sciences, National University of Medical Sciences (NUMS), Rawalpindi, Pakistan; ^3^Wuhan Institute of Biological Products Co., Ltd., Wuhan, Hubei, China

**Keywords:** Hantavirus, HFRS, HPS, immunotherapy, siRNA

## Abstract

Hantaviruses are a significant and emerging global public health threat, impacting more than 200,000 individuals worldwide each year. The single-stranded RNA viruses belong to the *Hantaviridae* family and are responsible for causing two acute febrile diseases in humans: Hantavirus pulmonary syndrome (HPS) and hemorrhagic fever with renal syndrome (HFRS). Currently, there are no licensed treatments or vaccines available globally for HTNV infection. Various candidate drugs have shown efficacy in increasing survival rates during the early stages of HTNV infection. Some of these drugs include lactoferrin, ribavirin, ETAR, favipiravir and vandetanib. Immunotherapy utilizing neutralizing antibodies (NAbs) generated from Hantavirus convalescent patients show efficacy against HTNV. Monoclonal antibodies such as MIB22 and JL16 have demonstrated effectiveness in protecting against HTNV infection. The development of vaccines and antivirals, used independently and/or in combination, is critical for elucidating hantaviral infections and the impact on public health. RNA interference (RNAi) arised as an emerging antiviral therapy, is a highly specific degrades RNA, with post-transcriptional mechanism using eukaryotic cells platform. That has demonstrated efficacy against a wide range of viruses, both *in vitro* and *in vivo*. Recent antiviral methods involve using small interfering RNA (siRNA) and other, immune-based therapies to target specific gene segments (S, M, or L) of the Hantavirus. This therapeutic approach enhances viral RNA clearance through the RNA interference process in Vero E6 cells or human lung microvascular endothelial cells. However, the use of siRNAs faces challenges due to their low biological stability and limited *in vivo* targeting ability. Despite their successful inhibition of Hantavirus replication in host cells, their antiviral efficacy may be hindered. In the current review, we focus on advances in therapeutic strategies, as antiviral medications, immune-based therapies and vaccine candidates aimed at enhancing the body’s ability to control the progression of Hantavirus infections, with the potential to reduce the risk of severe disease.

## 1. Introduction

Hantaviruses are negative-sense, single-stranded tri-segmented RNA viruses that belong to the order *Bunyavirales*, family *Hantaviridae* and genus *Orthohantavirus* (Kuhn and Schmaljohn, 2023). They exclusively maintain themselves in the population of their natural host and produce a persistent viral infection in them and make a continuous shedding in rodent excreta. They cause two acute febrile diseases in humans: Hantavirus pulmonary syndrome (HPS) and hemorrhagic fever with renal syndrome (HFRS) (Khaiboullina et al., 2005). Hantaviruses pose an emerging global threat to public health causing a devastating effect on human lives, affecting more than 200,000 individuals worldwide annually (Bi et al., 2008). Moreover, the number of cases is significantly increasing day by day in different parts of the world (Watson et al., 2014). Two major outbreaks of Hantavirus disease, reported in the last century, were the first to catch global attention. The first, HFRS outbreak, occurred during the Korean War (1950–1953), affected more than 3,000 US troops (Tian and Stenseth, 2019). The second, HPS outbreak, was documented in the southwestern regions of the US in 1993 (Mills et al., 1999). Pathophysiological studies have revealed that Hantavirus transmits by rodents, mainly through contaminated saliva, feces, urine, and aerosols. It can also be transmitted by bites of affected animals, though it is rarely reported (Brocato and Hooper, 2019). Although the Hantaviruses transmit from natural hosts to humans through a natural ecological process, the outbreak is accelerated by rodent animals like striped field mice and seasonal climatic fluctuations (Tian and Stenseth, 2019).

The clinical presentations of the disease depend on the geographic distribution of the viral strains around the world. In Asia, Hantavirus (HNTV) and Seoul virus (SEOV) primarily infect the human kidney and cause hemorrhagic fever with renal syndrome (HFRS). In North America, Andes virus (ANDV) and Sin Nombre virus (SNV) target the lungs and cause Hantavirus cardiopulmonary syndrome (HCPS) or Hantavirus pulmonary syndrome (HPS), with a high rate of mortality. While in Europe, Puumala virus (PUUV) and Dobrava-Belgarde virus (DOBV) cause a milder form of HFRS, the nephropathia epidemica (Echterdiek et al., 2019). These viruses, like any other enveloped virus, get inactivated when exposed to detergents, UV radiations, hypochlorite solutions, organic solvents, and high temperature (60°C for 30 min). They attach to the host cell surface receptors by glycoprotein and infect a number of cells including dendritic, lymphocyte, and epithelial cells. It has been revealed that integrins, the transmembrane proteins of the host cell, play a pivotal role in viral attachment and entry into the cell. Two types of integrin, β_1_ and β_3_-integrin interact with G_n_ of apathogenic Hantaviruses and glycoprotein of pathogenic Hantaviruses, respectively (Gavrilovskaya et al., 1998; Mackow and Gavrilovskaya, 2009).

## 2. Hantavirus genome organization

**Figure 1:**
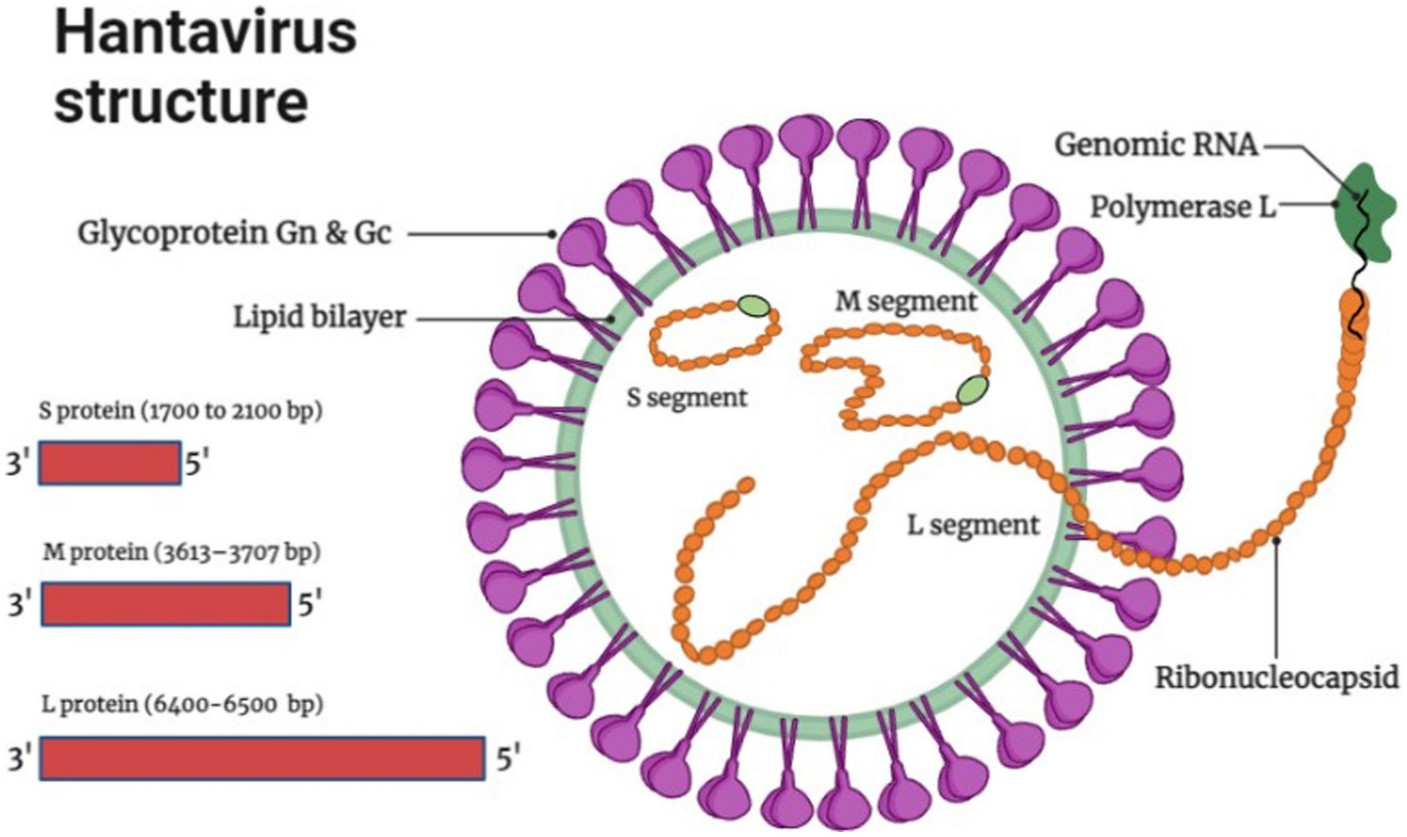
Hantavirus structure and genome organization (Image generated: www.biorender.com).

The genome of Hantaviruses contains three segments of single stranded, negative ribonucleic acid (RNA) molecules with a terminal sequence at their 3ˋend (Avšič-Županc et al., 2019) as shown in Figure 1. These genome segments are named according to their nucleotide sequence length as small (S), medium (M), and large (L) as shown in Figure 1 (Singh et al., 2022). Hantaviruses are enveloped viruses with a spherical structure of about 80 to 120 nm length in diameter. The envelope membrane is composed of bilayer of exterior lipids secreted from Golgi complex (Hepojoki et al., 2012). The lipid bilayer is lined with spikes (viral proteins) that protrude from the layer around 10 nm. These spikes appear as heterodimers of Gn and Gc glycoproteins and show a significant binding affinity with oligomers (Huiskonen et al., 2010). The complex and unusual symmetry of the spikes is considered rarely common in enveloped viruses (Huiskonen et al., 2010). The S segment comprises 1,700 to 2,100 bp nucleotides encoding N protein that synthesizes the nucleocapsid of the virion. The M segment containing 3,613–3,707 bp nucleotides encodes the envelop protein of 1,150 amino acids for glycoprotein. It carries a conserved pentapeptide motif “WAASA” at its N-terminus that acts as a target site for co-translational cleavage by the cellular peptidase complex (Löber et al., 2001). The L segment is 6,500 nucleotides wide and encodes about 2,160 amino acids for the biosynthesis of RNA-dependent RNA polymerase and other viral proteins like reverse transcriptase that converts the negative sense RNA into positive sense mRNA for protein synthesis and multiple genome copies. Variation and genetic alterations in M and S segments can disturb the virulence and antigenicity of virus (Du et al., 2014).

## 3. Prevalence

In humans Hantaviruses can cause serious fatal diseases such as hemorrhagic fever renal syndrome (HFRS) in Asia, HFRS and nephropathia epidemica (NE) in Europe and Hantavirus cardiopulmonary syndrome (HCPS) in North and South Americas. In recent years, almost 200,000 people around the world are affected by Hantaviruses annually, with a case fatality of 1–15% for HFRS, 0.1–1% for NE and up to 40–60% for HCPS. Bi et al. (2008) and Singh et al., (2022) two significant outbreaks were documented in the last century which brought Hantavirus disease to worldwide attention. More than 3,000 United Nations forces contracted the HFRS during the Korean War (1950–1953), which was the first time it happened and the second was the outbreak of HCPS in 1993 in the Four Corners area of the United States (Tian and Stenseth, 2019).

### 3.1. Asia

**Figure 2:**
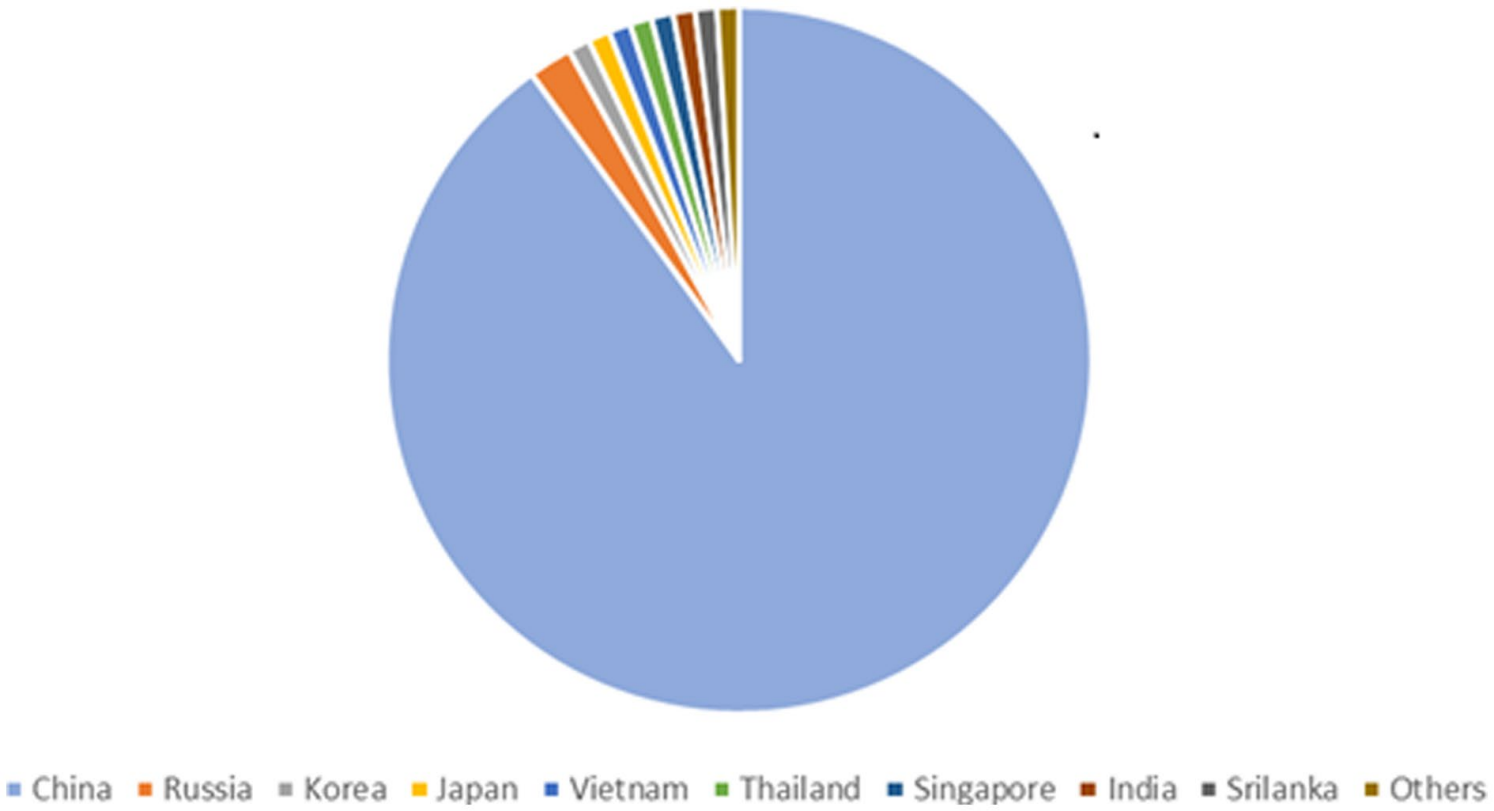
Epidemiology of Hantavirus cases across Asia, showing 90% infections in China, 2% in Russia and 1% in Korea, Japan, Vietnam, Thailand, Singapore, India, Srilanka, Others.

90% of all the documented cases worldwide are from China, which has the greatest frequency of HFRS, mainly caused by HTNV and SEOV. One of the key HFRS hotspots is the populous central Chinese province of Shaanxi (Yu et al., 2015). According to Chinese Center for Disease Control and Prevention (China CDC) a total of 77,558 cases and 866 fatalities were reported from 2006–2012 possessing a case fatality rate of 1.13%, death rate of 0.01 per 100,000 and a yearly incidence rate of 0.83 per 100,000 cases. HFRS cases have been documented in 30 out of 32 provinces in China as of yet (Zhang et al., 2014). HFRS cases have been mainly documented in autumn-winter and spring seasons (Ke et al., 2016). The incidence of HFRS in China remained low nationwide as compared to the previous five years. As of 18 December 2021, 29 provincial-level administrative divisions (PLDAs) reported 8,502 cases with 54 (0.63%) deaths which was 9.10 and 17.39% more than the 7,793 cases and 46 deaths in 2020 (Aitichou et al., 2005; Wei et al., 2021). Epidemiology of HTNV across asia is shown in Figure 2. The Khabarovsk region reported the first HFRS case in 1934 in Asian Russia. From 1978–1995 a total of 3,145 cases of HFRS in Asian Russia occurred, with a 1.7% morbidity rate (Onishchenko and Ezhlova, 2013). HTNV was first isolated in Korea and an average of 1% mortality rate is recorded for the 300–500 cases reported annually (Lee et al., 2013). A few HFRS have been reported in Vietnam (Huong et al., 2010), Thailand (Suputthamongkol et al., 2005), Singapore (Wong et al., 1985), Sri Lanka (Vitarana et al., 1988), India (Chandy et al., 2009) and Japan (Lokugamage et al., 2004) but more data needs to be recorded from these and other regions in Asia (Heyman et al., 2011).

### 3.2. Europe

**Figure 3:**
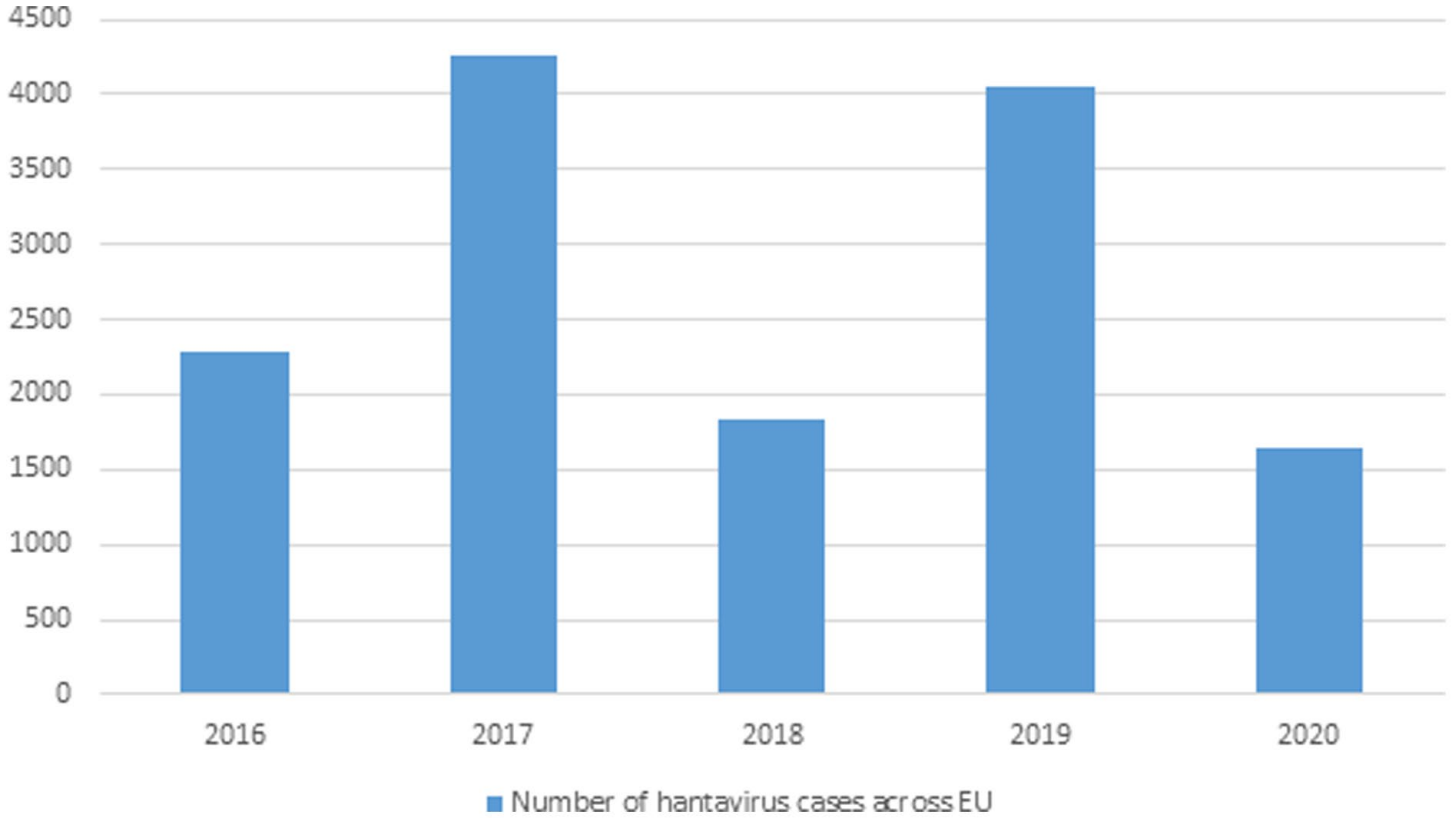
Data showing Hantavirus cases in the EU from 2016–2020 (This data was published by the European Center for Disease Control and Prevention).

Greater than 10,000 HFRS and thousands of NE (nephropathia epidemica) cases are identified on an annual basis all over Europe and are primarily caused by PUUV, DOBV, and SAAV (Vaheri et al., 2013) and (Jiang H. et al., 2017). HFRS cases have been recorded in Finland, Germany, Belgium, France, UK, Poland, the Balkans but in many other areas very few cases are recorded even though the sero-positive prevalence is high in them, which is why more data needs to be documented in Europe because Hantavirus associate infections are high in these areas but remain undocumented (Heyman et al., 2011). Greater than 2,800 cases of Hantavirus caused HFRS were reported in Germany in 2012 (Krüger et al., 2013). In UK SEOV virus associated with acute kidney injury (AKI) in rats was first isolated in Scotland in 1977 and only caused AKI in 1 of 15 rats (Duggan, 2019). Figure 3 No. of HTNV cases across Europe from 2016–2020.

### 3.3. Americas

In 1993, HCPS was initially identified as a hantaviral illness during the Four Corners outbreak in the USA. In North and South Americas almost 200 cases are documented annually. HCPS in Americas is primarily caused by ANDV and SNV. HCPS cases have also been reported in Bolivia, Paraguay, Uruguay, Brazil, Argentina, Panama, Chile, and Canada (Jonsson et al., 2010). In Canada 143 confirmed HCPS cases have been recorded as of 2020 (Warner et al., 2020). Each year between 100 and 200 cases of HCPS are recorded in Argentina, mostly in spring and summer (Jiang H. et al., 2017). Chile has the annual mortality rate of 32–35% for HCPS (Martinez-Valdebenito et al., 2014). Brazil recorded 2032 HCPS cases as of 2017. Table 1 shows the Hantavirus reported cases across North and South America (Warner et al., 2020; Armién et al., 2023).

**Table 1:** Reported HTNV cases across North, Central, and South America.

Country	Cases	Year	Source
USA	850	1993–2021	CDC
Canada	143	As of 2020	Warner et al. (2020)
Panama	712	1999–2019	Armién et al. (2023)
Costa Rica	3	Till 2016	PAHO
Argentina	1,350	As 2016	PAHO
Chile	1,028	As of 2016	PAHO
Brazil	2032	Till 2017	PAHO
Paraguay	319	Till 2016	PAHO
Uruguay	169	Till 2016	PAHO
Bolivia	300	Till 2016	PAHO
Ecuador	73	As of 2016	PAHO
Peru	6	As of 2016	PAHO
French Guiana	3	Till 2016	PAHO

### 3.4. Africa

No indigenous Hantavirus was recognized in Africa 15 years ago (Kruger et al., 2015). Since then, only a few studies have examined SEOV and other hantaviruses in Africa and their effects on human health. Due to the lack of regionally specific SEOV testing in human blood samples and the paucity of investigations, it appears that SEOV is not a significant public health hazard on the continent (Heyman et al., 2004). However, there are substantial indications that humans and wild rats in 17 different African nations may be infected with organisms similar to SEOV (Clement et al., 2019). In Africa, the SEOV is not seen as a serious hazard to the general population.

### 3.5. Pathogenesis

Hantaviruses infections cause HFRS, NE, and HCPS in humans, the symptoms associated with them and their mode of transmission is discussed below.

### 3.6. Symptoms associated with HFRS and NE

Kidneys are primarily affected in both HFRS and NE. NE is a relatively mild version of HFRS. Symptoms include thrombocytopenia, fever, differing degrees of acute renal failure, myalgia some cases have also reported symptoms related to ocular and central nervous systems. Both of them have five clinical phases febrile, hypotensive, oliguric, polyuric and convalescent. Complications can include multiorgan failure, bleedings, severe encephalomyelitis, pituitary hemorrhage, pulmonary edema, shock and fatal outcome in case of NC and all the aforementioned complications in addition to glomerulonephritis, respiratory distress syndrome and disseminated intravascular congestion can occur in HFRS as shown in Table 2 (Jiang et al., 2016; Jiang H. et al., 2017; Hautala et al., 2021).

**Table 2:** Depicts five phases of HFRS and NE and the main associated features.

Phase	Time of occurrence	Main features
Febrile	1–7 days	Myalgia, fever
Hypo-tensive	1–3 days	Hypo-tension
Oliguric	2–6 days	Decreased urine level
Polyuric	2 weeks	Increased urine level
Convalescent	3–6 months	Frailty, lethargy

### 3.7. Symptoms associated with HCPS

Lungs are mainly affected in HCPS. Three phases are associated with HCPS they are prodromal, cardiopulmonary, convalescent phases. Prodromal phase lasts 1–5 days and symptoms include malaise, fever, gastrointestinal distress and headaches. Cardiopulmonary phase symptoms include cardiopulmonary malfunction, pulmonary edema, cough, dyspnea and hypoxia. Convalescent phase is the recovery phase where all previous symptoms subside except for dyspnea, which can persist up to 1–2 years (Llah et al., 2018).

### 3.8. Spread of hantaviruses

Hantaviruses basically infect rodents and are also found in small insectivorous mammals and bats. They cause asymptomatic infections in rodents, and are transmitted to humans through rodent bite, inhalation of aerosolized virus particles and via inhalation of dried feces, urine and saliva of rodents. Hantavirus infection in humans mainly affects the endothelial cells in lungs and kidneys and leads to HCPS, NE, and HFRS (Ermonval et al., 2016). Environmental factors such as availability of food, climate change, geographical location also contributes to Hantavirus infection (Guterres and de Lemos, 2018).

### 3.9. Proposed mechanism of Hantavirus pathogenesis

**Figure 4:**
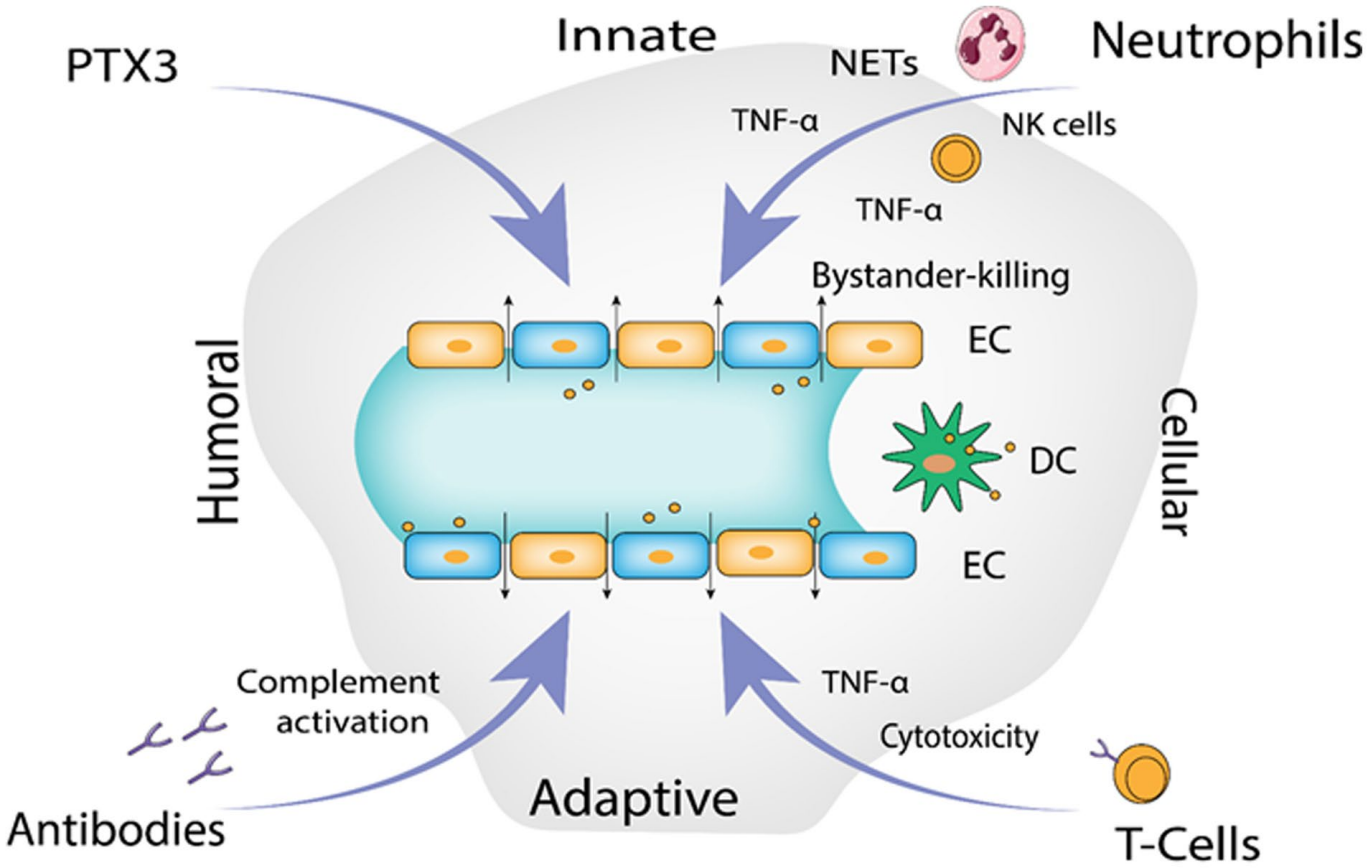
The proposed immunological mechanisms leading to endothelial barrier breakdown by the Hantavirus (Image generated: www.biorender.com).

The infection begins with contact of Gn and Gc surface proteins with β-integrin receptors, yet how hantaviruses spread in the human body is still not fully known. Given that they express 3-integrin receptors and are found close to epithelial cells, immature dendritic cells likely play a crucial role (Gavrilovskaya et al., 1999, 2002). Platelet dysfunction, immunological responses, and the disruption of endothelial cell barrier capabilities likely have a role in the pathological process of Hantavirus (Mackow and Gavrilovskaya, 2009). DCs in humans are extremely mobile, they link innate and adaptive immunity and reside in pathogen-host interface in the respiratory mucosa and lung alveoli. They have the ability to “snorkel” through the epithelial-tight junctions by introducing their dendritic projections into the airway lumen (Jahnsen et al., 2006) and contract Hantavirus in the lungs (Raftery et al., 2002; Marsac et al., 2011). Additionally, monocytes exposed to HTNV transform into cells that resemble DCs (Markotić et al., 2007; Schönrich et al., 2008) that might serve as Trojan horse, assisting viruses to spread across the human body and eventually infect endothelial cells in numerous organs. It has been seen in various studies that EC become stimulated during PUUV infection, increasing the production of chemokines and adhesion molecules such as E-Selectin, intercellular adhesion molecule 1 (ICAM-1) and VCAM-1 (Temonen et al., 1996). Stimulated chemokines include a neutrophil activator and recruiter called IL-8 (interleukin 8) (Klingström et al., 2008; Sadeghi et al., 2011; Libraty et al., 2012; Kyriakidis and Papa, 2013). Production of human leucocyte antigen mainly HLA-E is elevated on EC, which in turn activates nature killer (NK) cells (Kraus et al., 2004; Björkström et al., 2011). Hantavirus-infected EC may be removed by activated immune cell’s cytotoxic action, which could lead to vascular leakage (Hayasaka et al., 2007). HTNV infected EC are somewhat defended from cytotoxic T cells and NK cells (Gupta et al., 2013) uninfected EC’s are however, vulnerable to cytotoxic assault and bystander killing. According to recent studies, neutrophils can generally contribute to the immunopathogenesis (Gupta et al., 2010; Saffarzadeh et al., 2012) by interacting with active EC they go through 2 mechanisms of programmed cell death. Virus-induced 2 integrin signaling causes neutrophils to become activated, which then leads to either NETosis or the production of inflammatory cytokines like TNF-α or VEGF, depending on the 2 integrin ligands involved and possibly additional micro-environmental stimuli, resulting in increase in the vascular leakage, though through different processes (Schönrich et al., 2015). Pentraxin-related protein 3 (PTX3), a humoral pattern-recognizing receptor, activates complement during acute HFRS (Outinen et al., 2012). PTX3 is kept in neutrophil granules and released *via* integrins in response to signals (Jaillon et al., 2007; Razvina et al., 2015). In addition to cytoskeletal rearrangements in EC, the soluble complement components C3a and C5a produced after complement activation by antibodies and PTX3 also causes IL-8 production (Monsinjon et al., 2003). As a result, PTX3 draws additional neutrophils to the endothelium barrier, escalating the inflammation in the vessels. TNF alpha a pro-inflammatory cytokine is released by Hantavirus-infected macrophages, DC as well as NK cells, neutrophils and CD8+ T cells that have been activated (Raftery et al., 2002; Marsac et al., 2011; Shin et al., 2012). TNF-α (Tumor necrosis factor alpha) eliminates the virus from infected cells by non-cytolytic processes, it may, on the one hand, aid in the control of hantaviral propagation (Khaiboullina et al., 2000; Guidotti and Chisari, 2001). On the other hand, vascular leakage and breathing problems are generated if it is given externally in proportions that are encountered during Hantavirus infection (Tracey and Cerami, 1994; Wimer, 1998). Both direct and indirect methods may cause localized TNF-local release at the EC contact to promote vascular permeability(Schönrich et al., 2015). Figure 4 illustrates the proposed immunological mechanisms that result in endothelial barrier breakdown by the Hantavirus.

### 3.10. Replication cycle of hantaviruses

**Figure 5:**
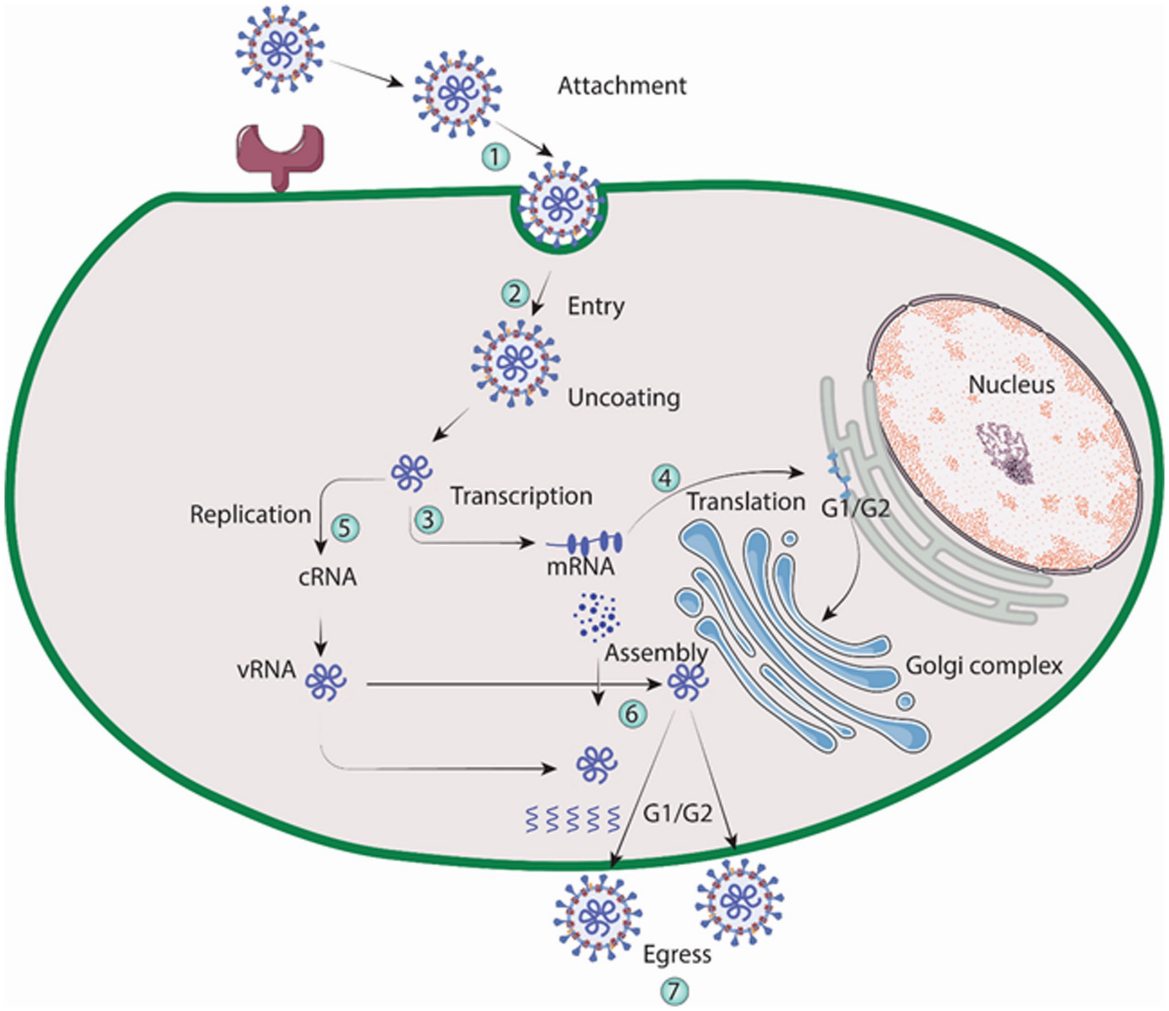
Replication cycle of Hantavirus (Image generated: www.biorender.com).

Macrophages and vascular endothelial cells, particularly those in the lungs and kidneys, are targeted by the Hantavirus (Yanagihara and Silverman, 1990). In order to facilitate binding, Gn protein of the virus engages with integrin receptors that reside on the outer layer of the cell it invades, according to a number of findings (Mir and Panganiban, 2010). A family of heterodimeric proteins called integrins include both alpha and beta chains. Both cell to extracellular matrix and cell to cell interactions are facilitated by it (Takagi and Springer, 2002; Campbell and Humphries, 2011). Virons are eventually carried to lysosomes after binding, which is accomplished *via* clathrin-coated pits. In the endolysosomal compartment, virions uncoat, releasing three viral nucleocapsids into the cytoplasm (Jin et al., 2002). The virus is engulfed by clathrin-coated vesicle (CCV), which is composed of clathrin-coated cellular membrane (Ramanathan and Jonsson, 2008). Three mRNAs transcribed by RdRp, one from the S, M, and L sections of the viral RNA. Free ribosomes are locations for the translation of the S and L derived mRNAs. Whereas, (RER) rough endoplasmic reticulum is where M-specific mRNAs are converted into proteins. Two glycoproteins, Gn and Gc, are produced as a result of the glycoprotein precursor’s intrinsic cleavage at a highly conserved amino acid sequence (Spiropoulou, 2001). For glycosylation, the Golgi complex receives the glycoproteins Gn and Gc, where hanta virions are thought to develop as illustrated in Figure 5. Followed by exocytosis, migration to the golgi cisternae, then to the outer membrane of secretory vesicles and finally egression through exocytosis. The details of virion egress, however, are mostly unclear (Szabo, 2017). Other possible mechanisms for virus entry include caveola, macropinocytosis, cholesterol-dependent endocytosis, clathrin-independent endocytosis-mediated receptor and micropinocytosis (Ramanathan et al., 2007).

## 4. Diagnosis

Initial diagnosis can be performed by observing the symptoms associated with HCPS, HFRS, and NE. Since Hantaviruses are rodent transmitted viruses so clues can be obtained for diagnosis at the time of taking patient’s history by asking if a patient recently traveled to areas infested with rodents or came in contact with rodents or their excretions. Screening tests such as Complete Blood Count (CBC) and peripheral smear can also be used (Dvorscak and Czuchlewski, 2014).

Serological methods available for definite diagnosis of infection caused by Hantaviruses. Serology is used to detect IgG and IgM anti-hantaviral antibodies in the blood of the patient. The IgM antibodies appear extremely early during the course of the infection, whereas IgG antibodies appear later in the process. Enzyme Linked Immunosorbent Assay (ELISA) and the strip immunoblot detects viral antibodies, only disadvantage is the potential for cross reacting with other viral antigens, but are still extremely useful and important for diagnosis purposes. Indirect Immunofluorescence assay is more specific than Elisa but it is laborious. Neutralization tests are the most specific serological tests but are costly, laborious and require BSL-3 conditions. Immunochromatographic tests are readily performed and are cheap but cross reactivity minimizes precision of results obtained (Schubert et al., 2001; Meisel et al., 2006; Navarrete et al., 2007).

Molecular methods include Real Time RT PCR, an important, sensitive and fast technique for detecting viral RNA in blood, blood clots or tissues, its only disadvantage includes that it only yields results during the viremic phase of the Hantavirus caused infection (Aitichou et al., 2005). Microarray technique is fast, sensitive and allows simultaneous detection of thousands of viruses but is costly and the data analysis is complex. Next Generation Sequencing (NGS) is also used for virus detection and complete sequencing but it is expensive and requires complex bioinformatics tools.

Virological methods include isolation in cell cultures which allows extensive functional and virological studies but is laborious and requires specially trained personnel and BSL-3 conditions. Immunohistochemistry by enzyme immunoassay and immunofluorescent test allows diagnosis from infected tissues of organs but laborious preparations are required (Kruger et al., 2015).

## 5. Treatment and management

Hantavirus infections are managed mostly by managing the symptoms, providing supporting care and admitting patients to the Intensive Care Unit (ICU), providing patients with oxygen therapy, by administrating an antiviral ribavirin, which has showed reduction in death rate of patients still in their initial stages of infection. Treatment for HCPS patients includes respiratory and cardiac monitoring and support which includes mechanical ventilation, hemofiltration and membrane oxygenation. Supportive treatment for HRFS includes electrolyte infusion and hydration to stabilize blood pressure, acute thrombocytopenia is managed with transfusing platelets, uremia is managed with intermittent hemodialysis and continuous renal replacement therapy is given to manage multi-organ pulmonary edema (Sargianou et al., 2012; Liu et al., 2020).

## 6. Recent therapeutic advances against Hantavirus

### 6.1. Blocking viral entry

The following candidate drugs have been shown to increase survival rate only in the initial stages of HTNV infection, but are ineffective in later stages.

#### 6.1.1. Griffithsin

A protein called griffithsin (GRFT), which was first discovered from red algae, has demonstrated potential as a multifunctional antiviral agent. Its capacity to prevent the invasion of several viruses, including HIV and SARS-CoV, has been researched (O'Keefe et al., 2010; Lusvarghi and Bewley, 2016). The spikes of the Hantavirus consist of tetramers formed by Gn-Gc heterodimers, which envelop the entire surface of the virus particle. Gn, one of the viral envelope glycoproteins, contains several N-linked glycosylation sites and is positioned on the virus surface, making it a potential primary target for GRFT. A high-mannose oligosaccharide-binding lectin called Griffithsin (GRFT) is now being tested in phase I clinical trials as a topical microbicide for the defense against several viruses. It is a powerful inhibitor of ANDV infection. The fact that GRFT prevented the entry of pseudo-particles containing ANDV envelope glycoprotein into host cells suggests that it prevents the function of viral envelope protein during entry. To combat ANDV and SNV infection, 3mGRFT (trimeric synthetic tandemer of GRFT) is more effective than GRFT (Shrivastava-Ranjan et al., 2020). In a recent study GFRT prevented the entry and HTNV infection of recombinant vesicular stomatitis virus (VSV) containing HTNV glycoproteins into host cells *in vitro* by attaching to the viral N-glycans. It also shielded the suckling mice from death brought on by cerebral exposure to HTNV, according to *in vivo* tests. These findings highlighted the importance of GRFT as a potential HTNV infection inhibitor (Zhao et al., 2022).

#### 6.1.2. Coumarin

Coumarin and its derivatives have been found to have antiviral activity against a variety of viruses, including human immunodeficiency virus (HIV), hepatitis virus, herpes simplex virus, CHIKV, and enterovirus 71. Coumarin derivative’s potential use is as antiviral medicines. The diverse chemical makeup of coumarin derivatives however, meant that these substances had an impact on the many stages of a virus life cycle. Tricyclic coumarin GUT-70 inhibited HIV’s ability to attach and fuse to cellular walls and plasma membranes, while dipyranocoumarin (+)-calanolide A could block reverse transcription (Xu et al., 2021). Coumarin dimmer analogs could also block HIV integrase, and amide coumarin derivatives could affect how HIV was put together. On the basis of molecular structure of coumarin, two of the most common coumarin derivatives, dicoumarin and pyrone-coumarin, were synthesized. It was demonstrated that the tri-fluoro substituent on the benzene ring of the dicoumarin derivatives N6 and N7 had a strong anti-HTNV action (Li et al., 2022a). Dicoumarin demonstrated increased anti-HTNV activity, and adding Cl or CF3 might increase the inhibitory activity and selectivity to the HTNV, according to the structure activity relationship (SAR). To clarify the connection between the chemical structure and the biological action against the HTNV, more research is necessary. N6, a derivative of coumarin, showed both *in vivo* and *in vitro* action against HTNV, and AKT1 may have played a role in the molecular mechanism by which N6 combats viral infection (Reguera et al., 2010; Li et al., 2021, 2022b). Therefore, finding novel coumarin compounds that could fight viral infection has significant significance for creating potent drugs.

#### 6.1.3. Lactoferrin

Lactoferrin (LF), a glycoprotein that binds to iron and has been shown to have broad antibacterial, antifungal and antiviral properties, inhibited hantaviral attachment, adsorption (Masson et al., 1969; Buys et al., 2011). Both *in vitro* and *in vivo* studies have demonstrated that LF guards against Hantavirus infection (Murphy et al., 2001). Lactoferrin was found partially effective in preventing HFRS by restricting focus formation in suckling mice, but when used *in-vivo* in combinations with ribavirin, it prevented focus development entirely (Murphy et al., 2001).

In a study, Seoul virus (SEOV) was used to infect Vero E6 cells in order to test the antiviral potency of LF against hantaviruses. The objective of the study was to contrast LF’s antiviral activity with that of Rbv.100 μg/mL of RBV administered after infection reduced the number of foci by 97.5%. Vero E6 cells treated with 400 μg/mL of LF showed a significant 85% decrease in the number of foci as compared to the control group. However, in LF-pretreated cells, the number of foci began to increase by 24 h post-infection (hpi). At 24 hpi LF prevented viral shedding, but not after 48 hpi (Murphy et al., 2001). As shown by another supporting study, the findings suggested that LF may attach to cell surfaces and prevent SEOV from adhering to host cells in the early stages of infection (Murphy et al., 2000). It is interesting to note that, LF improved cell survival rates following hantaviral amplification despite not inhibiting the formation of NP or Gc. This is due to the ability of LF to increase the cytocidal activity of NK cells (Murphy et al., 2001). However, the specific method through which LF block SEOV absorption, affect host immune responses and has an effect on other Hantavirus species are still unknown.

#### 6.1.4. Virus fusion inhibitors (domain III and stem peptides)

Several processes are thought to be involved in virus-cell membrane fusion (Kielian and Rey, 2006; Harrison, 2015). During viral fusion, fusion proteins become activated and insert a fusion peptide or loop into the target membrane. As a result, an intermediate phase is generated in which one end of the fusion protein can join to the viral envelope *via* transmembrane region, linking the viral and cellular membranes together. The fusion protein undergoes conformational changes which can draw both anchors together, achieving a hairpin-like structure where both domains gather at one end. After that, the outer leaflets of membranes fuse to form a hemi-fusion intermediate, followed by complete membrane fusion once the opposed membranes have been brought together with the help of local membrane curvature. A pore is formed as a result of the fusion, allowing the virus to inject its ribonucleocapsids into the cytoplasm of the cell and begin replication.

On the basis of molecular structures, viral fusion proteins are categorized into three classes: I, II, and III. Alpha helices make up the majority of class I fusion proteins, while beta sheets make up the majority of class II proteins. Class III fusion proteins exhibit characteristics from both class I and class II (Kielian, 2014; Modis, 2014; Harrison, 2015). *In silico* and *in vitro* investigations reveal that Hantavirus Gc glycoprotein resembles class II fusion proteins (Cifuentes-Munoz et al., 2011). Three domains (I-III) and a stem connecting the ectodomain to the transmembrane region make up class II fusion proteins (Rey et al., 1995; Kielian, 2006). During fusion, DIII advances toward the fusion loop to adopt a hairpin-like structure (Bressanelli et al., 2004; Gibbons et al., 2004). This movement is followed by stem region which folds against the trimeric core created by the fusion protein (Roman-Sosa and Kielian, 2011; DuBois et al., 2013; Klein et al., 2013). It is possible to intervene and stop the fusion process as a result of these significant conformational changes, thereby inhibiting viral infection. It is possible to delay or block viral entry by selectively binding ligands to a fusion protein’s intermediate form before it assumes its post-fusion conformation (Barriga et al., 2016). It has been demonstrated that protein fragments spanning the stem region and domain III (DIII) are capable of inhibiting these fusion proteins. Due to this, to block viral fusion and entrance into the cell, recombinant ANDV DIII and stem peptides were developed. A 60% reduction in Vero E6 infection by ANDV *via* the endosomal pathway was achieved by combining DIII with the C-terminal of stem region. Over 95% infection was prevented when ANDV fused at the plasma membrane. These findings indicated that a stem fragment method used against Hantavirus may obviously block the fusion of related viruses belonging to the same genus (Barriga et al., 2016).

#### 6.1.5. Hantavirus-binding receptor inhibitors (cyclic nonapeptides)

Hantaviruses have two transmembrane glycoproteins, Gn and Gc, derived from a single glycoprotein precursor through proteolytic cleavage that occurs during post-translational modifications (Jonsson and Schmaljohn, 2001). The entry of hantaviruses into human endothelial cells is facilitated by the interaction of viral surface glycoproteins with αvβ3 integrins present on the host cell surface (Gavrilovskaya et al., 1999; Raymond et al., 2005). Both Gn and Gc may play a role in the entry of viruses, Gn is involved in the viral attachment process while Gc is thought to drive membrane fusion (Mackow and Gavrilovskaya, 2001; Tischler et al., 2005).

Small molecules like peptide ligands have potential therapeutic applications as they can bind to specific proteins and interfere with particular protein–protein interactions (Mayor et al., 2021). For this purpose, particular peptides can be synthesized either by imitating one of the binding partners or by creating new binding interactions. Novel peptide ligands were created with the ability to block SNV infection. SNV, the causative agent of HCPS, is classified as a category A pathogen by the NIAID. Currently, there is no specific treatment for HCPS and its fatality rate remains high reaching approximately 40%.

Therapeutics that target SNV particularly are still lacking. To tackle this issue, researchers are utilizing phage display technique to discover cyclic nonapeptides that can bind to the cellular receptor αvβ3. By identifying these peptides, they aim to prevent Hantavirus entry including SNV into the human endothelial cells particularly during *in vitro* experiments with Vero E6 cells (Larson et al., 2005; Hall et al., 2007). These peptides offer therapeutic promise while avoiding the potential adverse effects often associated with conventional mAbs employed as therapeutic agents to inhibit such interaction (Baudouin et al., 2003; Gauvreau et al., 2003). Cyclic nonapeptides were created using peptide sequences from phage that displayed the most potent infection inhibition. However, when tested, the isolated peptides showed lower effectiveness in blocking infection (ranging from 9.0 to 27.6% inhibition) compared to the phage-presented peptides, which achieved inhibition levels of 74.0 to 82.6%. As the phage displayed pentavalent peptides, the focus was on exploring whether presenting the identified peptides in a multivalent manner would lead to enhance inhibition. In order to do this, certain cyclic peptides were bound to multivalent nanoparticles using carboxyl linkages and their ability to suppress infection was examined (Hall et al., 2008).

With a 4:1 nanoparticle-to-virus ratio, SNV infection was reported to be inhibited *in vitro* by two of the synthetic cyclic nonapeptides CLVRNLAWC and CQATTARNC. CLVRNLAWC inhibited the infection by 9–32.5% while CQATTARNC inhibited it by 27.6–37.6%. At a 20:1 ratio, CQATTARNC decreased infection by 50% (Hall et al., 2008). These findings demonstrate the potential therapeutic value of multivalent inhibitors in the disruption of interactions between proteins, particularly those critical for host cell viral infection. Based on molecular makeup and potential capacity to engage the αvβ3 cell receptor, further peptidomimetic compounds were selected. In the first round of screening, 49 peptidomimetic compounds and in the second round, 68 compounds were found having an anti-Hantavirus action in lower 2,000 μM range. Due to this, a special collection of chemicals were acquired for the subsequent phases of drug development. The antiviral potential of these chemical compounds requires improvement and *in vivo* research to support it (Hall et al., 2010).

### 6.2. Blocking viral replication

#### 6.2.1. Ribavirin

**Figure 6:**
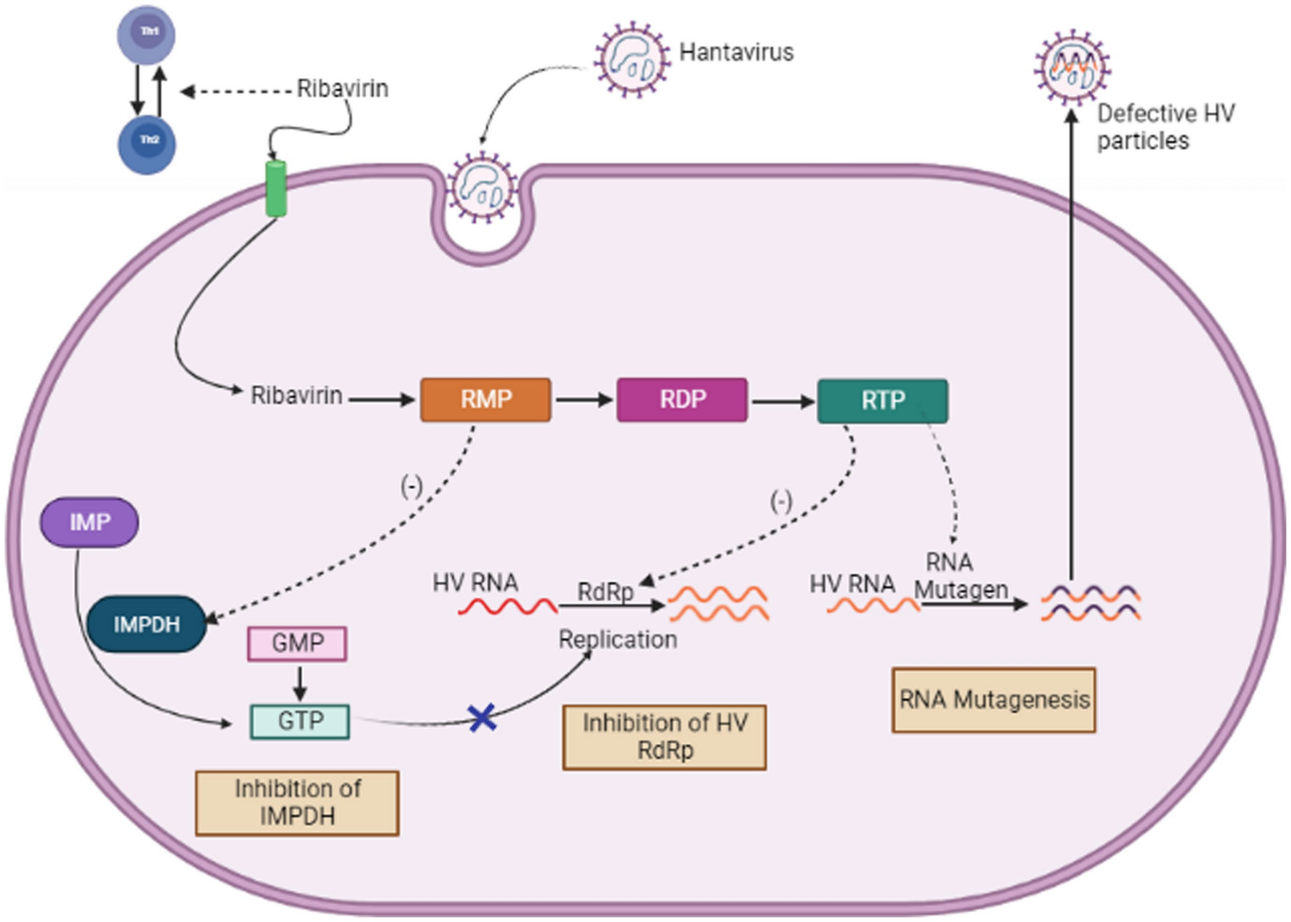
Mechanism of Action of Ribavirin (Image generated: www.biorender.com).

When used both *in vitro* and *in vivo*, the purine nucleoside analog ribavirin exhibits a wide range of antiviral effects against numerous different RNA, DNA viruses and it works by blocking viral replication. However, its pleiotropic effects, make it difficult to understand how it works (Fernandez-Larsson and Patterson, 1990; Graci and Cameron, 2006). These contain both direct mechanisms, like interfering with RNA capping, inhibiting polymerase activity, and inducing lethal mutagenesis, as well as indirect mechanisms, such as inhibiting inosine monophosphate dehydrogenase and exerting immunomodulatory effects (see Figure 6). The first specialized phase in the cellular production of guanine nucleotides, involves the catalytic activity of enzyme IMPDH. This process relies on NAD^+^ and involves the transformation of inosine monophosphate to xanthosine monophosphate. Because Ribavirin 5′-monophosphate (RMP) bears structural resemblance to GMP, It serves as a highly effective competitive inhibitor of IMP dehydrogenase (Streeter et al., 1973). Human IMP dehydrogenase (IMPDH) type I and type II isoforms are both potently inhibited by RMP, which reduces *de novo* GTP production. Because of this disturbance, viral RdRp (RNA-dependent RNA polymerase) cannot function properly. The antiviral effects of ribavirin are assumed to be related to its ability to inhibit IMP dehydrogenase (IMPDH) (Graci and Cameron, 2006).

Moreover, RBV was found to control host immunological responses by inhibiting the production of interleukin-10 by regulatory T cells (Kobayashi et al., 2012). The blocking mechanism of ribavirin is believed to target the capping (Goswami et al., 1979) and translation efficiency (Toltzis and Huang, 1986) of viral mRNA, along with the direct reduction of the viral polymerase’s activity (Wray et al., 1985; Fernandez-Larsson and Patterson, 1989). Ribavirin acts by interfering with the accuracy and effectiveness of polymerase replication. As a result, it may induce chain termination more frequently or improper nucleotide insertion, which could result in error catastrophe. Recent investigation showed that ribavirin rises the chances of error made by the Hantavirus polymerase within a cell culture model (Severson et al., 2003). We assumed that this elevated error rate is because of the integration of ribavirin into the viral RNAs *via* RNA-dependent RNA polymerase. It has been shown that ribavirin causes hantaviruses to replicate in an error-prone manner, which lowers the viral titer (Severson et al., 2003).

Although more investigation is required to completely understand how ribavirin inhibits Hantavirus, the findings of Severson et al. (2003) indicated that ribavirin may function as a mutagen *via* directly integrating into the S-segment of cRNA or mRNA through the viral RdRp. The amount of error-free mRNA falls when ribavirin is integrated into mRNA, which might lower the amount of viral proteins required to construct infectious viral particles. The amount of mRNA was, however, significantly decreased in these experiments. This finding implies that the ribavirin integration may reduce mRNA stability. This theory appears to be a logical and convincing explanation given the virus life cycle. As a result, the integration of ribavirin could accelerate viral mRNA to become more unstable and break down more quickly within the host cell (Jonsson et al., 2005).

#### 6.2.2. ETAR

ETAR, also known as (1-beta-d-ribofuranosyl-3-ethynyl-[1,2,4] triazole), functions as a nucleoside analog and is similar to ribavirin in that it prevents HV replication by lowering the levels of GTP. Notably, ETAR showed superior efficacy to ribavirin as a therapeutic option in trials on suckling mice infected with HTNV. Due to the absence of pseudo base pairs, ETAR is unlikely to cause mutations (Chung et al., 2008). Furthermore, there is no proof that ETAR has any immunoregulatory effects (Szabo, 2017).

#### 6.2.3. Favipiravir

T-705 is a pyrazine derivative also referred to as favipiravir or 6-fluoro-3-hydroxy-2-pyrazinecarboxamide. It was initially known for its ability to combat influenza, but it is now thought to have antiviral capabilities against a number of other viruses that rely on RdRp for replication (Furuta et al., 2009). Flaviviruses, noroviruses, arenaviruses, and bunyaviruses (Gowen et al., 2007) are some of these viruses. T-705 works as a prodrug that is transformed into T-705-4-ribofuranosyl-5-triphosphate with the help of several intracellular enzymes. When T-705-4-ribofuranosyl-5-triphosphate is created, it functions as a purine nucleotide analog, it is incorporated into the newly synthesized RNA chain, selectively inhibiting RdRp (Furuta et al., 2005). Favipiravir causes the production of non-infectious viral particles when it is employed by the viral polymerase as an alternative nucleoside substrate.

Favipiravir was investigated in both non-fatal and fatal SNV/ANDV hamster models, and the outcome showed a reduction in viral load in hamster serum and different organs. Furthermore, in the lethal ANDV infection model, the use of Favipiravir led to 100% survival (Safronetz et al., 2013). Other studies have shown that after viremia has started, the ANDV/hamster model did not offer protection against delayed antiviral treatment (Munir et al., 2021).

#### 6.2.4. Baloxavir acid

Small molecules also gained importance for their antiviral potential (Deng et al., 2020). Baloxavir, a recently licensed influenza medication derived from BXM, is an endonuclease-targeting small molecule that prevents the translation of the influenza virus and also prevents the virus from replicating. Since the mRNA produced by the viral RdRp lacks a 5′ cap and both hantaviruses and influenza viruses fall within the category of negative-sense RNA viruses, these viruses must acquire a host mRNA cap and apply it to their mRNA (Reguera et al., 2010). BXA specifically targets the PA-PB1-PB2 trimer belonging to the endonuclease domain of the influenza virus. The structures of the RdRp molecules of VSV, a mononegavirus, and another bunyavirus, La Crosse virus (LACV), among others, suggest that the RdRp molecules of negative-sense viruses share certain fundamental characteristics. Particularly within the order *Bunyavirales*, the endonuclease domain of RdRp appears to be more conserved than the other domains. BXA was incorporated into the active center of the RNA endonuclease domain by utilizing the well-known ANDV LP RNA endonuclease domain structure and computationally modeling the HTNV domain. This novel method revealed a previously unknown relationship with Influenza B virus (IBV), illuminating the potential fitness implications. Modeling outcomes might explain why BXA inhibits Hantavirus replication. Additionally, because of the structural closeness of the endonucleases in these viruses, this approach may also apply to arenaviruses (Ye et al., 2019).

#### 6.2.5. siRNA-based therapy

**Figure 7:**
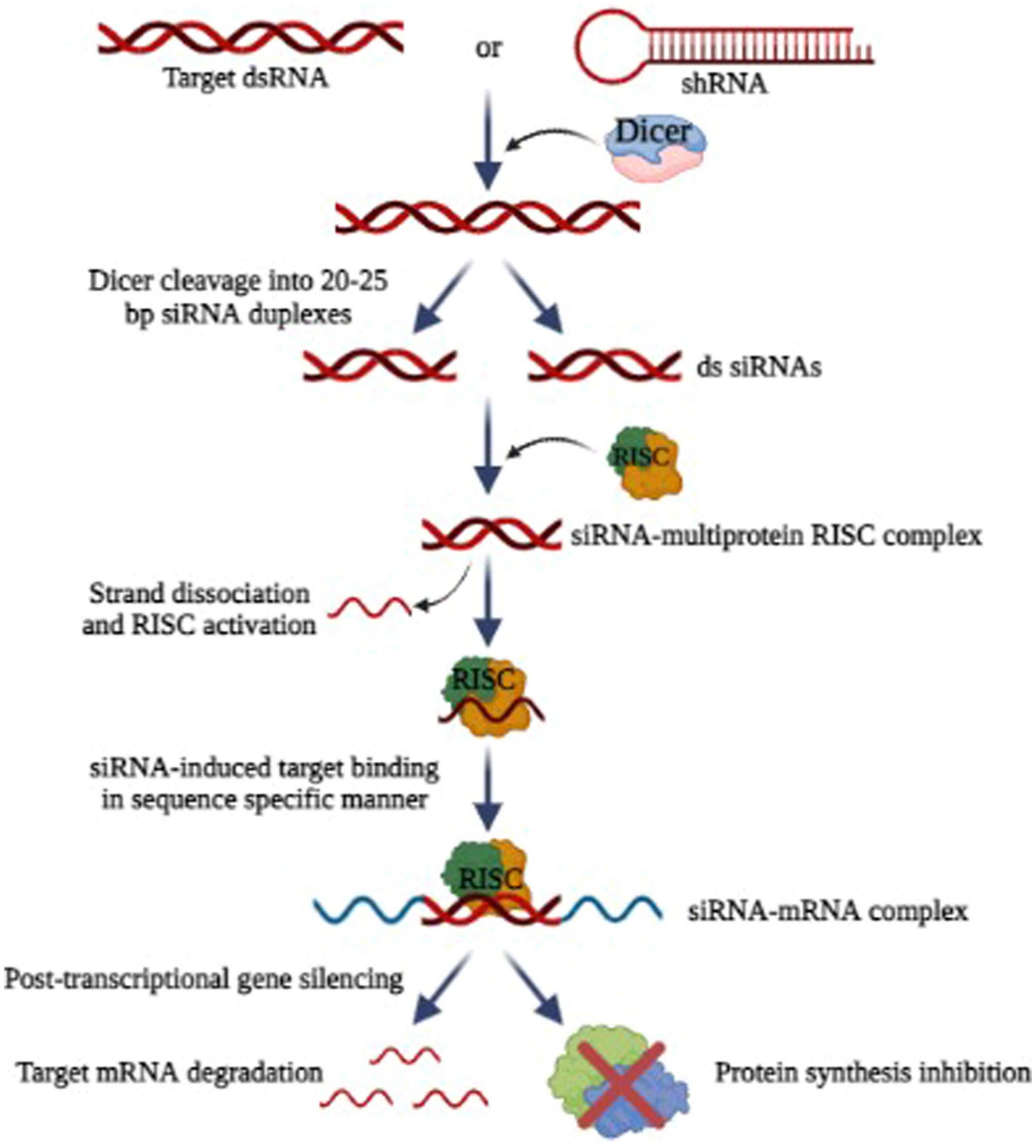
Mechanism of RNA interference (Image generated: www.biorender.com).

RNA interference (RNAi) has emerged as a new exciting frontier for antiviral therapies. RNAi is a post-transcriptional, and highly specific cellular mechanism in eukaryotic cells where small non-coding RNA molecules (typically 21–25 nucleotides long) bind to specific mRNA molecules and degrade them, thereby inhibiting the expression of specific genes (Ambesajir et al., 2012). For the degradation and cleavage of the mRNA before translation, the siRNA duplexes are integrated into an RNA-induced silencing complex (RISC) (Fire et al., 1998; Elbashir et al., 2001; MacRae et al., 2006). The siRNAs possess complementary sequences that bind to the target mRNA after transcription and inhibit its translation, as illustrated in Figure 7. The precision and accuracy of RNAi make it an accessible option for gene silencing (Tian et al., 2021). Since the discovery of RNAi in 1900, RNAi technologies have developed rapidly to suppress rogue viruses. So, there is a wide range of viruses that can be inhibited by RNAi-based methods, both *in vivo* and *in vitro* (Weinberg and Arbuthnot, 2010).

Research has shown that HIV-1, polioviruses, nairoviruses, and Lassa viruses can be eradicated using RNAi as a strategy *in vitro* by inhibiting viral replication (Flusin et al., 2011). Researchers found that RNAi can be used to inhibit SARS-CoV-2 replication (Ricci et al., 2020). For some other viruses that affect humans and animals, RNAi-based therapies effectively reduce viral loads and increase the chances of survival (DeVincenzo et al., 2010). But the problem with RNAi technology is its delivery and most of the studies are only limited to *in vitro*. Scientists are working to find carriers to safely deliver the siRNA (Bobbin and Rossi, 2016). In this way, RNAi combinations can avoid problems associated with multidrug sensitivities and toxicity. By targeting rapidly evolving viral sequences, RNAi-based therapies prevent the emergence of drug-resistant viruses (Chen et al., 2018). It is also possible for RNAi-based drugs to produce a sustained therapeutic response since genes can be introduced.

Hantaviral replication can be prevented most directly and effectively by targeting viral RNAs. A prospective antiviral method has been evaluated using siRNA against targeted hantaviral genes, which may enhance virus RNA clearance based on RNA interfering (RNAi) processes. In Vero E6 cells or human lung microvascular endothelial cells, it has been demonstrated that siRNAs targeting the S, M, or L segments of the ANDV may decrease viral replication. In Vero E6 cells, an S-targeted siRNA pool appeared to be more efficient in inhibiting viral transcription and replication than an M- or L-targeted siRNA pool (Chiang et al., 2014). Significantly, even if administered after infection, these siRNAs may prevent ANDV replication.

However, since siRNAs are low in biological stability and *in vivo* targeting ability, their antiviral efficacy may be severely hindered despite their successful inhibition of Hantavirus amplification in host cells. One method of treating in a mouse model of HTNV-induced encephalitis is intraperitoneal administration of recombinant antibodies that recognize HTNV Gc (3G1-Ck-tP). These antibodies were combined with siRNAs that target the encoding regions of the HTNV genome. This resulted in siRNAs being delivered precisely to HTNV-infected brain cells and HTNV intracranial infection being prevented (Yang et al., 2017). Moreover, the shRNA expression showed promising results by inhibiting HTNV infection in both *in vitro* and *in vivo* (Liu et al., 2016). To ensure the stability and selectivity of siRNAs, innovative delivery mechanisms should be created; however, it is yet unknown how effective and safe these systems will be in the treatment of HFRS or HCPS.

### 6.3. Host-targeting antiviral

#### 6.3.1. Vandetanib

Hantavirus-induced increased endothelial microvascular cell permeability was checked through *in-vitro* techniques. The expression of the cellular adhesion molecules including VE-cadherin and VEGF were both increased and decreased as a result of ANDV infection, which also increased the phosphorylation of the VEGF-receptor 2 (VEGFR2) (Gorbunova et al., 2010; Bird et al., 2016). As a result of ANDV infection, the VEGF-A expression was shown to be enhanced in the 3D model of human lungs tissue (Sundström et al., 2016).

The activation of SFK (Src family kinases) signaling may result from the binding of VEGF to VEGFR2, which has the ability to cause the dissociation, internalization, and destruction of VE-cadherin. The structural integrity of adherent junctions was damaged as a result of changes in VE-cadherin expression and localization, which led to an increase in cellular permeability (Jiang et al., 2016). According to studies, the connection between β3 integrin and VEGFR2 can be disrupted by HTNV or ANDV infection, which causes excessive phosphorylation of VEGFR2. Because of this disturbance, infected endothelium cells may become more permeable by becoming more VEGF-responsive (Wang et al., 2012).

In one investigation, it was discovered that the use of a VEGFR2 kinase inhibitor and SFK inhibitors significantly reduced the increased endovascular permeability brought on by ANDV. Particularly successful were the SFK inhibitors dasatinib and pazopanib, which prevented VE-cadherin separation by more than 90% (Gorbunova et al., 2011). Another study demonstrated that Vandetanib, a tyrosine kinase inhibitor specifically target VEGFR2, has a capacity to inhibit *in vitro* phosphorylation of VEGFR2, leading to the reduction of VE cadherin degradation (Bird et al., 2016). However, Vandetanib showed signs of potential side effects during human studies, including hypertension, dermatologic responses, and other cardiorespiratory consequences (Grande et al., 2013).

In the ANDV/hamster model, giving the therapy at doses of 10, 25 and 50 mg/kg/day, beginning 5 days prior the ANDV threat, resulted in a slowed mortality and raised overall survival rate by 23%. The same tiny therapeutic molecules, however, failed to protect the hamsters from a lethal ANDV challenge when given once viremia had started in the hamster model infected with ANDV (Munir et al., 2021).

#### 6.3.2. Bradykinin B2 receptor antagonists

Using bradykinin receptor antagonists as a treatment for Hantavirus infections is another interesting strategy. All Hantavirus infections commonly cause vascular leakage and increased capillary permeability. The underlying mechanisms that result in alterations in vascular permeability following Hantavirus infection are yet unknown. Hantaviruses have been reported to be the cause of enhanced stimulation of kinin-kallikrein system following endothelial cells infection, which leads to release of bradykinin (Golias et al., 2007). Bradykinin is a nonapeptide-binding bradykinin B2 receptor that functions as an inflammatory mediator that causes vessels to dilate, increases vascular leakage and lowers blood pressure in Hantavirus infection.

It is acknowledged as the main facilitator of vascular leakage by destroying inter-endothelial connections. Additionally, the synthesis of interleukin-1 and tumor necrosis factor alpha is also induced (Maurer et al., 2011). Icatibant, a synthetic polypeptide that resembles bradykinin structurally, works as a strong, focused, and aggressive antagonist of the bradykinin B2 receptor. Icatibant binds to the bradykinin B2 receptor, preventing bradykinin from attaching to this receptor (Taylor et al., 2013). It stops vasodilatation brought on by bradykinin in humans and in C1 esterase inhibitor-knockout animals, it reverses enhanced vascular permeability, as well as inhibiting bradykinin-induced effects *in vivo* with dose- and time-dependent inhibition (Cockcroft et al., 1994; Cicardi et al., 2010). Following subcutaneous injection, Icatibant is almost completely bioavailable. Many people just need a single 30-mg dose because the drug is well tolerated (Cicardi et al., 2010; Deeks, 2010).

### 6.4. Corticosteroid therapy/anti-inflammatory agents

As discussed already, Hantavirus affects endothelium cells and causes the host to produce pro-inflammatory cytokines especially TNF-α during the course of infection. Although not categorized as an antiviral, restricting this aspect of the immunological response to virus infection was hypothesized to offer potential clinical advantages. An immunomodulatory treatment was firstly performed in which only intramuscular injection or oral administration of cortisone was permitted. Although there was no reduction in mortality with this therapeutic approach, fatality was decreased to shock. An additional method to administer methylprednisolone for the treatment of HCPS was used but the outcome offered no clinical advantages (Priya and Priya, 2020).

### 6.5. Immunotherapy

Immunotherapy could be done against HTNV by utilizing neutralizing antibodies (NAbs) generated as a result of Hantavirus infection. It has been established that human convalescent plasma offers protective benefits to animals in both infection and lethal disease models of SNV/mouse (Medina et al., 2007) and ANDV/hamster (Brocato et al., 2012), respectively. These findings determine the sufficiency of neutralizing antibodies in preventing infection and disease (Brocato and Hooper, 2019).

Studies have only been done with animal models (suckling mice, hamsters, infant rats) where antibody efficacy was measured at cellular level using focus reduction neutralization test (FRNT) and hemagglutination test (HI) (Manigold and Vial, 2014). Although presently no particular effective treatment has been found against hantaviruses that cause HPS, according to numerous studies neutralizing antibodies can block HPS *in vivo*. An open trial has been carried out in Chile to assess the effectiveness of using human immunological sera as an HPS therapy (Vial et al., 2015). When compared to the 32% case fatality rates in the rest of the nation over the course of the study, the results showed a borderline statistical significance (Vial et al., 2015; Dheerasekara et al., 2020).

It was hypothesized that treatment at an earlier stage of HTNV infection could further enhance results. However, the practical implementation of immunotherapy is restricted by the requirement for ABO blood typing of convalescent plasma and the absence of a standardized product, which prevents its widespread use (Brocato and Hooper, 2019).

#### 6.5.1. Monoclonal antibodies

Antibodies have been employed on a global scale to prevent and treat viral infectious diseases (Casadevall, 1999). Researchers are currently working on generating antibodies targeting various viruses responsible for causing hemorrhagic fevers. One instance involves the development of a group of engineered human monoclonal antibodies (MAbs) designed to combat the Ebola virus (Maruyama et al., 1999). These specialized antibodies have shown efficacy not only in treating infections but also in providing protection before and after exposure to various viruses, including cytomegalovirus (CMV) (Aulitzky et al., 1991) and respiratory syncytial virus (RSV) (Malley et al., 1998). Up until now, there has not been a successful therapeutic antibody available for clinical use in treating and preventing Hantavirus infection. As a result, the development of Hantavirus-neutralizing MAbs is of utmost importance to establish effective immunotherapy and prophylaxis against Hantavirus infection (Xu et al., 2002).

During the 1980s, researchers extensively studied monoclonal antibodies targeting HTNV, detailing their interactions with the glycoproteins, Gn and Gc, and identifying specific neutralization sites (Arikawa et al., 1989). In an attempt to establish a link between particular viral epitopes and protection against HTNV infection, researchers conducted assessments on monoclonal antibodies using passive transfer techniques (Schmaljohn et al., 1990). This significant experiment provided compelling evidence that out of the 15 tested monoclonal antibodies, a neutralizing immune response to either Gn or Gc alone is adequate to prevent HTNV infection in hamsters. Following the HTNV/hamster experiment, the effectiveness of the recombinant Human GP monoclonal antibody HCO2 in providing protection was verified (Liang et al., 1996). This protective potential of neutralizing monoclonal antibodies was further validated in a later study utilizing a suckling mouse/HTNV model (Arikawa et al., 1992).

In a recent development, two genetically engineered monoclonal antibodies demonstrated their ability to protect hamsters from fatal ANDV-HPS (Garrido et al., 2018). One source patient with high antibody titers was chosen after screening 27 ANDV convalescent HCPS sera. Utilizing recombinant DNA technology, researchers generated recombinant monoclonal antibodies from memory B cells specific to the ANDV glycoprotein. The resulting candidates, JL16 and MIB22, displayed effective neutralization of ANDV *in vitro* (Garrido et al., 2018; Dheerasekara et al., 2020). When passively transferred antibodies on days 3 and 8 after infection, the administered antibodies successfully prevented lethality in hamsters infected with ANDV via intranasal exposure, whether given individually or in combination (Brocato and Hooper, 2019).

In another investigation, researchers produced and analyzed 18 murine monoclonal antibodies (MAbs) targeting HTNV strain Chen. Out of these, 13 MAbs were directed at the viral nucleocapsid protein (NP), four identified the viral envelope glycoprotein G2, and one MAb responded with both NP and G2. Only the monoclonal antibodies (MAbs) that specifically targeted epitopes on G2 exhibited positive results in the hemagglutination inhibition (HI) test. Additionally, these MAbs demonstrated *in vitro* virus-neutralizing activity and provided *in vivo* protection against HTNV infection in susceptible mice. Because the mice received virus-neutralizing MAbs one day before and two days after being exposed to HTNV, all of them were protected. This suggests that these particular neutralizing MAbs could be helpful for both pre- and post-exposure preventive measures, as well as potential immunotherapy against HTNV infection. In endemic regions of China, Phase II clinical trials are being conducted to evaluate the efficacy of these neutralizing MAbs as an emergent treatment for patients in the early stages of HRFS (Xu et al., 2002).

Anti-Hantaan Virus murine monoclonal antibody was developed for treating HFRS and a single dose of 2.5-20 mg was administered to healthy Chinese volunteers intravenously and the results indicated that it was nicely received (Xu et al., 2009). The cross-reactivity of novel and previously created MAbs effective against N protein of TULV, TMPV, DOBV and PUUV were assessed contrary to N proteins of fifteen shrew and rodent borne hantaviruses using various immunological techniques in order to have a large collection of well-described Hantavirus-specific MAbs. The results indicated that all MAbs, with the exception of those that are exclusive to TPMV, displayed various cross-reactivity patterns with Hantavirus N proteins and recognized native viral antigens in infected mammalian cells (Avižinienė et al., 2023). A recently discovered widely neutralizing antibody was structurally analyzed by Mittler et al. (2023) on a patient who had recovered from Puumala virus (Old world Hantavirus) infection. The authors created an improved variant of this patient-extracted antibody that could defend against Andes (New world Hantavirus) and Puumala virus, in rodent models using functional research, structural data along with complementary binding and neutralization. The therapeutic candidate ADI-65534, which is a broadly neutralizing antibody possesses a potential to treat Hantavirus infections.

#### 6.5.2. Polyclonal antibodies

In a recent study, polyclonal immunotherapy in which purified IgG polyclonal antibodies produced in DNA immunized alpacas (ANDV M/SNV M) were given to Syrian hamsters, which protected them completely against HPS (Sroga et al., 2021). Studies have demonstrated the immunogenicity of Hantavirus DNA vaccines in various animal species, such as geese, rabbits and ducks (Hooper et al., 1999; Brocato et al., 2012, 2013). A substantial step forward was recently achieved with the development of a fully human polyclonal antibody using trans-chromosomal bovine that had received ANDV and SNV DNA vaccines. This product has demonstrated positive protection against two deadly HPS animal models (Hooper et al., 2014). Presently, researchers are actively working to broaden their investigation and develop a standardized polyclonal antibody capable of targeting a variety of Hantaviruses, including HTNV, ANDV, SNV, and PUUV. The objective is to advance this product through preclinical testing and ultimately carry out a Phase 1 clinical study, paving the path for potential therapeutic applications. Table 3 (Vilcek, 1991; Murphy et al., 2000, 2001; Sundstrom et al., 2001; Glass et al., 2003; Maes et al., 2004; Chung et al., 2008, 2013; Hall et al., 2008; Antonen et al., 2013; Ogg et al., 2013; Safronetz et al., 2013; Vial et al., 2013, 2015; Laine et al., 2015; Barriga et al., 2016; Bird et al., 2016; Garrido et al., 2018; Brocato and Hooper, 2019).

**Table 3:** Lists some examples of potential antiviral therapies against Hantavirus.

Antiviral therapy	Type	Function	Target	Disease	References
Lactoferrin	Lactoferrin	Block viral entry	Viral GP	HFRS	Murphy et al. (2000, 2001)
Ribavirin	Nucleoside analogs	Inhibit viral replication	RdRp	HCPS and HFRS	Chung et al. (2013) and Ogg et al. (2013)
Favipiravir	Pyrazine derivatives	Block viral entry	RdRp	HCPS	Safronetz et al. (2013)
Vandetanib	Tyrosine kinase inhibitor	Improve vascular function	VEGF/Vascular function	HCPS	Bird et al. (2016)
ETAR	Nucleoside analog	Inhibit viral entry	RdRp	HCPS and HFRS	Chung et al. (2008)
Corticosteroids	Hormone	Rebuild immune homeostasis	Immunotherapy	HCPS and HFRS	Vial et al. (2013) and Brocato and Hooper (2019)
Human Immune Sera	Human pAbs	Block viral entry	Viral GP	HCPS	Vial et al. (2015)
JL16 and MIB22	Human mAbs	Block viral entry	Viral GP	HCPS	Garrido et al. (2018)
Domain III and stem peptides	Peptides	Block viral entry	Gc glycoprotein	HCPS and HFRS	Barriga et al. (2016)
CLVRNLAWC and CQATTARNC	Cyclic nonapeptides	Block viral entry	Host receptor	HCPS	Hall et al. (2008)
Icatibant	Small molecule	Improve vascular function	BK type 2 receptor	HFRS	Antonen et al. (2013) and Laine et al. (2015)
TNF-α	Small proteins/Pro- inflammatory cytokines	Increase systemic toxicity	Vascular function	HCPS and HFRS	Vilcek (1991), Sundstrom et al. (2001) and Maes et al. (2004)
RANTES/IP- 10/MCP-1	Small proteins/Pro-inflammatory chemokines	Immunomodu lators/Inhibit viral infection	Microvascular endothelium	HFRS	Sundstrom et al. (2001) and Glass et al. (2003)

## 7. Vaccines and immunotherapy

As viruses are constantly emerging from zoonotic origin, vaccines seem to be the most effective therapeutic option to reduce the incidence of disease (Ahmed et al., 2022). Different vaccines are under development against hantaviruses to improve protective efficacy and safety profiles (Saavedra et al., 2021). Attenuated and killed vaccines are the most common and primitive method of vaccine development that is injected into the animal or human body to elicit a protective immune response (Naeem et al., 2021). These vaccines are prepared by growing the isolated viral strain on the Vero cell line followed by inactivation through physical and chemical means. Similarly, the formalin-inactivated vaccine, Hantavax, was the first developed vaccine to prevent hantaviral infection in South Korea, it was developed using the HTNV strain ROK 84/105, which multiplies in lactating mice’s brains. Its clinical trial proved that it was well endured in human volunteers and successfully lowered the incidence of HFRS patients (Cho et al., 2002; Dheerasekara et al., 2020). However, the neutralizing antibody response was poor after two doses, therefore 3rd dose was injected to attain protective immune response in the host that lasts for 3–4 year (Dheerasekara et al., 2020). A bivalent inactivated vaccine against infection caused by SEOV and HTNV was developed in 1994 and was approved for use in China in 2005. It was found effective against HTNV and SEOV infections (Cho et al., 2002).

The success achieved by Hantavax is to decrease the incidence of hantaviral infection (Munir et al., 2021) but still needs a more effective and safe vaccine against Hantavirus which becomes possible to improve through modernization in virology and molecular biology with the development of more applied biological techniques. Hantavax is less efficient for long-term immunity and negligible cell-mediated immunity which can be overcome by immunizing the individual multiple times. Therefore, a vaccine is required that induces more effective and long-lasting immunity against the Hantavirus (Mohsen et al., 2017). Virus-Like Particles are considered efficient with better safety profiles, and prolonged immunity with the production of high titers of antibodies in humans (Mohsen and Bachmann, 2022). VLPs of Hantavirus are constructed using the M and S gene segment or only the M segment that interacts with each other to form virus-like particles *in vitro* similar to Hantavirus virion (Acuña et al., 2014; Brocato and Hooper, 2019) along with the incorporation of CD40L or GMCSF gene segment in vectors that stimulates activation of macrophages and dendritic cells (Ying et al., 2016; Dong et al., 2019). CD40L or GMCSF decorated VLPs provide prolonged immunity with elevated humoral and cell-mediated immune response than undecorated VLPs (Ying et al., 2016; Dong et al., 2019). Another approach involves the insertion of gene segments from nucleocapsid protein from DOBV, HTNV, and PUUV into HBV core particles, it is shown to be highly immunogenic with or without adjuvant that stimulates the generation of all classes of IgG antibodies (Brocato and Hooper, 2019).

Recombinant vaccines are constructed using Glycoprotein C (Gc), Glycoprotein N (Gn), or Nucleocapsid protein that shows high immunogenicity and antigenicity, bearing the ability to induce protection against Hantavirus (Dheerasekara et al., 2020). The baculovirus expression system was used to develop the more efficient recombinant vaccines using Gc, Gn, or N protein (Dheerasekara et al., 2020) followed by immunization in hamsters which develop partial protection from infection when used solely either Gc or Gn and complete protection when Gc/Gn used in combination or immunized with N protein (Brocato and Hooper, 2019). The nucleocapsid protein is more conserved among different hantaviral species, therefore, the immune response produced against N protein induces highly cross-reactive antibody responses to PUUV, DOBV, and ANDV produced by *E. coli* (Krüger et al., 2011). Moreover, the use of adjuvants enhances the immunogenicity and protective efficacy of the vaccine in humans (Porter et al., 2012).

The S and M cDNA segment of HTNV was inserted into the vaccinia virus to develop a molecular virus vector-based vaccine (Brocato and Hooper, 2019). Protective efficacy was evaluated in Syrian hamsters by inoculating two doses of VACV-vectored HTNV vaccine which resulted in protection from HTNV or SEOV but not PUUV (Munir et al., 2021). Cross-reactive antibodies of HTNV protected against SEOV but were unable to protect from PUUV (Malinin and Platonov, 2017). Clinical trials were conducted to further evaluate the vaccine efficacy (McClain et al., 2000; Brocato and Hooper, 2019) and it was confirmed that the vaccine was safe to inoculate and resulted in the production of neutralizing antibodies against both VACV and HTNV, and the subcutaneous route was preferred to administer the vaccine (Perley et al., 2020). The efficacy of the vaccine was also evaluated in vaccinated and non-vaccinated individuals and 72% efficacy was observed in non-vaccinated and 26% in vaccinated individuals (Dheerasekara et al., 2020). Moreover, non-replicating adenovirus vectors were used to provoke vigorous cytolytic response when ANDV N protein, Gn, Gc, or Gc and Gn in combination was expressed (Safronetz et al., 2009). Similarly, the Vesicular stomatitis virus (VSV) pseudo-type virus containing Gn and Gc of Hantavirus and ANDV glycoprotein precursor (GPC)was separately inoculated in mice and hamsters and it produced robust neutralizing antibody response (Saavedra et al., 2021). However, it required 3 doses to produce long-term immunity in an individual (Brocato and Hooper, 2019; Saavedra et al., 2021).

All vaccines have shown their efficacy against hantaviruses but the safety and efficacy was varied between primitive and modern-generation vaccines. DNA vaccine encoding the HTNV Gn and lysosomal-associated membrane protein 1 which directed to major histocompatibility complex II (MHC) and processed as an exogenous antigen resulting in robust humoral and cell-mediated immune response (Jiang D.-B. et al., 2017). Normally, inactivated vaccines have poor immunological memory but DNA vaccines have much-improved memory profiles. The S and M segments of SEOV were cloned into an expression vector pWRG7077 or Sindbis replicon vector (Munir et al., 2021). The results depicted that the M segment had shown improved protection from SEOV infection in Syrian hamsters (Brocato et al., 2021). Therefore, the M segment of HTNV, PUUV, ANDV, SNV, SEOV, and DOBV is used for vaccination in non-human primates that elicits an elevated level of antibody response (Liu et al., 2020). Several DNA vaccines against HTNV associated infections are currently undergoing clinical trials. Hopper’s group have developed several vaccines against the envelope glycoprotein gene of hantaviruses and further studies have confirmed the ability of these vaccines to produce neutralizing antibodies against HFRS in multiple animal species and even protected hamsters against HFRS (Schmaljohn et al., 2014). Apart from vaccine development, delivery methods and routes are much more significant to achieve desired efficacy. Multiple administration routes are studied and found gene gun inoculation is more effective than any other route (Dheerasekara et al., 2020). Moreover, a combination of different DNA vaccines of hantaviruses is much more efficient to provoke an immune response rather than a single vaccine administration (Brocato et al., 2013). Currently no FDA approved treatment and vaccines are available against HTNV worldwide. There are still many unresolved issues regarding mass production, safety and efficacy and no significant effect in lowering the severity of the disease. However, more research is required in vaccine delivery routes to improve the immunogenicity of the vaccine.

Apart from vaccines, monoclonal and polyclonal antibodies are used to eliminate hantaviral pathogens in the human or animal body against Gc and Gn (Duehr et al., 2020). Furthermore, DNA vaccination of ANDV, SNV, HTNV, and PUUV in bovines produces purified polyclonal human IgG antibodies exhibiting high neutralization activity and provided effective protection against lethal HCPS (Liu et al., 2020). Therefore, it is worldwide tested and used as a promising prophylactic therapy. The administration of neutralizing antibodies during the acute phase of HPS is considered an effective treatment for hantaviral infection (Iglesias et al., 2022). The patients with low titers of neutralizing antibodies often had a severe disease while mild disease cases were present in individuals with higher antibody titers (Iglesias et al., 2022; Iheozor-Ejiofor et al., 2022). Therefore, different clinicians and scientists speculated that a strong neutralizing antibody response or passive immunization can efficiently reduce the severity of the disease by reducing viremia (Shah et al., 2020).

## 8. Future therapeutic developments

### 8.1. Hantavirus-induced cytokine and chemokine response

One of the main contributors to HPS and HFRS symptoms during the course of Hantavirus infection may be the cytokine production. Cytokines, particularly TNF-α, IL-1, and IL-6 play a vital role in inducing fever and septic shock. TNF-α is produced by Hantavirus infected neutrophils, NK cells, CD8+ T cells, DC and macrophages (Schönrich et al., 2015). The excessive production of TNF-α may cause systemic toxicity (Maes et al., 2004). Although the precise mechanism is yet unknown, these cytokines also play a significant part in the vascular permeability seen in HPS and HFRS (Sundstrom et al., 2001). One of the most significant pro-inflammatory cytokines is TNF-α. Monocytes and macrophages infiltrate the area of inflammation and release TNF-α (Vilcek, 1991). Patients with severe NE were found to have significantly higher plasma levels of TNF-α (Linderholm et al., 1996). The kidney biopsies from NE patients revealed that TNF-α expression was elevated in the peritubular regions (Temonen et al., 1996). Patients with HTNV infection have higher serum levels of soluble IL-2 receptor (sIL-2R) and soluble IL-6 receptor (sIL-6R). sIL-2R and sIL-6R serum levels increased two days and six days after the onset of HFRS, respectively (Markotic et al., 2002). The infected monolayers of endothelial cells remained irreversibly permeable while uninfected monolayers fully restored their function (Niikura et al., 2004). Chemokines serve as inflammatory mediators and are responsible for the regulation of viral infections. HTNV infected endothelial cells *in vitro*, resulted in the production of significant amount of interferon-inducible protein (IP-10) and regulated upon activation, normal T cell expressed and secreted (RANTES), also called CCL5 (Sundstrom et al., 2001). Another study showed production of chemokines like RANTES, IP-10, IL-6, and IL-8 by HTNV but NYV failed to generate the majority of these cellular chemokines (Geimonen et al., 2002). Inflammatory chemokines like RANTES and monocyte chemotactic protein-1 (MCP-1), usually produced by acute respiratory viruses, can increase inflammatory responses giving rise to virus immunopathology (Glass et al., 2003). All supernatant cell lines harboring hantaviruses have a substantial increase in RANTES mRNA (Khaiboullina and St. Jeor, 2002). The conclusion that chemokines play a significant role in virus pathogenesis, which has been drawn for other viruses, is supported by this data. Due to the dual functions that these chemokines play in viral infections, inhibiting them or using them as immunomodulators may be important strategies to treat or reduce viral illness, depending on the type of virus. During HTNV infection, production of IP-10 and RANTES can lead to an increased effector immune response directed against the infected vascular endothelial cells (Sundstrom et al., 2001). Consideration should be given to a general therapeutic strategy for Hantavirus infections that combine antiviral and anti-chemokine therapy.

### 8.2. Potential vaccines undergoing development against Hantavirus

Hantaviruses enter human societies by zoonotic transmission *via* inhaling contaminated aerosols. Normally, half a million people are infected worldwide annually with a mortality rate of up to 40%. The HFRS and HPS seem to be serious health threats in endemic areas due to their cryptic transmission and unpredictable nature of disease occurrence in healthy adults with elevated case fatality rates. The inappropriate commercialization of therapeutics in endemic areas constantly increases the prevalence of the disease. Moreover, no special antiviral drugs were found efficient to be used in hantaviral infection except ribavirin. However, during HPS and HFRS cases, the ribavirin had been found effective but its efficacy could not prevent the severity of the disease.

Since there are so many cases of hantaviral infection each year, medicinal countermeasures to prevent infection from these viruses must be developed. Animal models have repeatedly shown that antivirals are not effective if administered after the onset of viremia. Therefore, the development of vaccination and an antiviral that can be used separately or in combination is much necessary for public health. Immunization may provide long-lasting immunity while an antiviral, such as polyclonal antibody treatment, would provide immediate immunity. So, vaccines and passive immunotherapy can effectively prevent and treat hantaviral infections in endemic regions of the world. The transmission from person to person becomes limited by vaccination and some vaccines are currently undergoing clinical trials (see Table 4) (Hooper et al., 2001, 2006, 2013; de Carvalho et al., 2002; Geldmacher et al., 2004; Maes et al., 2006, 2008; Safronetz et al., 2009; Brocato et al., 2013; Prescott et al., 2014; Jiang D.-B. et al., 2017; Dong et al., 2019; Khan et al., 2019; Warner et al., 2019).

**Table 4:** Describing evaluation of Hantavirus vaccines in various animal models and some vaccines currently undergoing clinical trials.

Vaccine type	Antigen	Animal model	Immunogenicity evaluation	References
Inactivated vaccine	Formalin inactivated HNTV	Humans	Humoral response Neutralizing antibodies	Khan et al. (2019)
Virus-like particles	HTNV-VLP with CD40L or GM-CSF	Mice	Cytotoxic response Neutralization antibody Cytolytic activity	Dong et al. (2019)
	M	DHFR-deficient CHO cells	Antigen-specific IFN-γ production Effective against HTNV Still in developing phases	Dong et al. (2019)
Virus-vector vaccines	Replication-competent VSV-vectored SNV or ANDV glycoproteins	Syrian Hamster	Cross-reactive IgG antibodies Neutralizing antibodies	Warner et al. (2019)
	Replication-competent VSV-vectored ANDV glycoproteins	Syrian Hamsters	Neutralizing antibodies	Prescott et al. (2014)
	Non-replicating Ad vector expressing N, Gn, Gc, or Gn/Gc	Syrian Hamsters	CD8+ cell response Neutralizing antibodies	Safronetz et al. (2009)
Recombinant vaccines	Yeast-expressed DOBV nucleoprotein	Mice	NP-specific IgG response Th1/Th2 response Cross-reactivity with HTNV and PUUV	Geldmacher et al. (2004)
	Nucleoproteins from ANDV, TOPV, DOBV or PUUV	Bank voles	Specific CD8+ cell production Cross-reactive response against PUUV	de Carvalho et al. (2002)
	Truncated recombinant PUUV nucleoprotein linked to bacterial membrane protein	Mice	CD8+ T-cell response NP IgG response	Maes et al. (2006)
DNA vaccines	HTNV/PUUV/SNV/ANDV M gene segment mix	Rabbits	Neutralizing antibodies	Hooper et al. (2013)
	HTNV M segment	Rhesus macaques	Neutralizing antibodies Cross-reactivity with SEOV and DOBV	Hooper et al. (2001)
	ANDV and HNTV M gene segments	Rhesus macaques	Neutralizing antibodies	Hooper et al. (2006)
	SNV M gene segment	Syrian hamsters	Neutralizing antibodies	Hooper et al. (2013)
	PUUV M gene segment	Syrian hamsters	Protection against lethal ANDV infection, without nAbs Neutralizing antibodies	Brocato et al. (2013)
	Gn glycoprotein	BALB/c mice	Effective against HTNV Still in developing phases	Jiang D.-B. et al. (2017)
Subunit vaccines	NP (nucleocapsid protein)	E.coli mutant ICONE NMRI mice	Effective against PUUV In developing Phases	Maes et al. (2008)

## Author contributions

LA and SA conceived the study. LA, MA, MH, MS, AA, AB, JY, and NK searched the literature and drafted the manuscript. LA, SA, AB, NK, and JY critically reviewed the manuscript. All authors contributed to the article and approved the submitted version.
