# Corrigendum: Hantavirus: an overview and advancements in therapeutic approaches for infection

**DOI:** 10.3389/fmicb.2023.1343080

**Published:** 2023-12-12

**Authors:** Samia Afzal, Liaqat Ali, Anum Batool, Momina Afzal, Nida Kanwal, Muhammad Hassan, Muhammad Safdar, Atif Ahmad, Jing Yang

**Affiliations:** ^1^CEMB, University of the Punjab, Lahore, Pakistan; ^2^Department of Biological Sciences, National University of Medical Sciences (NUMS), Rawalpindi, Pakistan; ^3^Wuhan Institute of Biological Products Co., Ltd., Wuhan, Hubei, China

**Keywords:** hantavirus, HFRS, HPS, immunotherapy, siRNA

In the published article, there were errors in Table 1, Table 3, and Table 4.

The caption for Table 1 only listed North and South America, but Table 1 contains countries from North, Central, and South America.

In Table 1, the reference for the row “Canada” was incorrectly listed as “Jonsson et al. (2010)”. The correct reference is “Warner et al. (2020)”.

In Table 1, the reference for the row “Panama” was incorrectly listed as “Martinez-Valdebenito et al. (2014)”. The correct reference is “Armién et al. (2023)”. The corrected Table 1 and its caption “Reported HTNV cases across North, Central, and South America.” appear below.

**Table 1:** Reported HTNV cases across North, Central, and South America.

**Country**	**Cases**	**Year**	**Source**
USA	850	1993–2021	CDC
Canada	143	As of 2020	Warner et al. (2020)
Panama	712	1999–2019	Armién et al. (2023)
Costa Rica	3	Till 2016	PAHO
Argentina	1,350	As 2016	PAHO
Chile	1,028	As of 2016	PAHO
Brazil	2,032	Till 2017	PAHO
Paraguay	319	Till 2016	PAHO
Uruguay	169	Till 2016	PAHO
Bolivia	300	Till 2016	PAHO
Ecuador	73	As of 2016	PAHO
Peru	6	As of 2016	PAHO
French Guiana	3	Till 2016	PAHO

In Table 3, the reference for the row “Lactoferrin” was incorrectly listed as “Gorbunova et al. (2010) and Arikawa et al. (1989)”. The correct reference is “Murphy et al. (2000, 2001)”.

In Table 3, the reference for the row “Ribavirin” was incorrectly listed as “Schmaljohn et al. (1990) and Liang et al. (1996)”. The correct reference is “Chung et al. (2013) and Ogg et al. (2013)”.

In Table 3, the reference for the row “Favipiravir” was incorrectly listed as “Arikawa et al. (1992)”. The correct reference is “Safronetz et al. (2013)”.

In Table 3, the reference for the row “Vandenatib” was incorrectly listed as “Garrido et al. (2018)”. The correct reference is “Bird et al. (2016)”.

In Table 3, the reference for the row “ETAR” was incorrectly listed as “Golias et al. (2007)”. The correct reference is “Chung et al. (2008)”.

In Table 3, the reference for the row “Coritcosteroids” was incorrectly listed as “Mills et al. (1999) and Xu et al. (2009)”. The correct reference is “Vial et al. (2013) and Brocato and Hooper (2019)”.

In Table 3, the reference for the row “Human Immune Sera” was incorrectly listed as “Tian et al. (2021)”. The correct reference is “Vial et al. (2015)”.

In Table 3, the reference for the row “JL16 and MIB22” was incorrectly listed as “Weinberg and Arbuthnot (2010)”. The correct reference is “Garrido et al. (2018)”.

In Table 3, the reference for the row “Domain III and Stem Peptides” was incorrectly listed as “Taylor et al. (2013)”. The correct reference is “Barriga et al. (2016)”.

In Table 3, the reference for the row “CLVRNLAWC and CQATTARNC” was incorrectly listed as “Cicardi et al. (2010)”. The correct reference is “Hall et al. (2008)”.

In Table 3, the reference for the row “Incatibant” was incorrectly listed as “Avižiniene et al. (2023) and Mittler et al. (2023)”. The correct reference is “Antonen et al. (2013) and Laine et al. (2015)”.

In Table 3, the reference for the row “TNF-α” was incorrectly listed as “Brocato et al. (2012), Manigold and Vial (2014), and Vial et al. (2015)”. The correct reference is “Vilcek (1991), Sundstrom et al. (2001), and Maes et al. (2004)”.

In Table 3, the reference for the row “RANTES/IP-10/MCP-1” was incorrectly listed as “Manigold and Vial (2014), and Malley et al. (2004)”. The correct reference is “Sundstrom et al. (2001) and Glass et al. (2003)”. The corrected Table 3 and its caption “Lists some examples of potential antiviral therapies against Hantavirus.” appear below.

**Table 3:** Lists some examples of potential antiviral therapies against Hantavirus.

**Antiviral therapy**	**Type**	**Function**	**Target**	**Disease**	**References**
Lactoferrin	Lactoferrin	Block viral entry	Viral GP	HFRS	Murphy et al. (2000, 2001)
Ribavirin	Nucleoside analogs	Inhibit viral replication	RdRp	HCPS and HFRS	Chung et al. (2013) and Ogg et al. (2013)
Favipiravir	Pyrazine derivatives	Block viral entry	RdRp	HCPS	Safronetz et al. (2013)
Vandetanib	Tyrosine kinase inhibitor	Improve vascular function	VEGF/Vascular function	HCPS	Bird et al. (2016)
ETAR	Nucleoside analog	Inhibit viral entry	RdRp	HCPS and HFRS	Chung et al. (2008)
Corticosteroids	Hormone	Rebuild immune homeostasis	Immunotherapy	HCPS and HFRS	Vial et al. (2013) and Brocato and Hooper (2019)
Human Immune Sera	Human pAbs	Block viral entry	Viral GP	HCPS	Vial et al. (2015)
JL16 and MIB22	Human mAbs	Block viral entry	Viral GP	HCPS	Garrido et al. (2018)
Domain III and stem peptides	Peptides	Block viral entry	Gc glycoprotein	HCPS and HFRS	Barriga et al. (2016)
CLVRNLAWC and CQATTARNC	Cyclic nonapeptides	Block viral entry	Host receptor	HCPS	Hall et al. (2008)
Icatibant	Small molecule	Improve vascular function	BK type 2 receptor	HFRS	Antonen et al. (2013) and Laine et al. (2015)
TNF-α	Small proteins/Pro- inflammatory cytokines	Increase systemic toxicity	Vascular function	HCPS and HFRS	Vilcek (1991), Sundstrom et al. (2001) and Maes et al. (2004)
RANTES/IP- 10/MCP-1	Small proteins/ Pro-inflammatory chemokines	Immunomodulators/ Inhibit viral infection	Microvascular endothelium	HFRS	Sundstrom et al. (2001) and Glass et al. (2003)

In Table 4, the reference for the row “Inactivated Vaccine” was incorrectly listed as “Sroga et al. (2021)”. The correct reference is “Khan et al. (2019)”.

In Table 4, the reference for the row “Virus-like Particles 1” was incorrectly listed as “Jonsson et al. (2005)”. The correct reference is “Dong et al. (2019)”.

In Table 4, the reference for the row “Virus-like Particles 2” was incorrectly listed as “Wray et al. (1985)”. The correct reference is “Dong et al. (2019)”.

In Table 4, the reference for the row “Virus-Vector Vaccines 1” was incorrectly listed as “Hopper et al. (1999)”. The correct reference is “Warner et al. (2019)”.

In Table 4, the reference for the row “Virus-Vector Vaccines 2” was incorrectly listed as “Brocato et al. (2013)”. The correct reference is “Prescott et al. (2014)”.

In Table 4, the reference for the row “Virus-Vector Vaccines 3” was incorrectly listed as “Deng et al. (2020)”. The correct reference is “Safronetz et al. (2009)”.

In Table 4, the reference for the row “Recombinant Vaccines 1” was incorrectly listed as “Hopper et al. (2014)”. The correct reference is “Geldmacher et al. (2004)”.

In Table 4, the reference for the row “Recombinant Vaccines 2” was incorrectly listed as “Ogg et al. (2013)”. The correct reference is “de Carvalho et al. (2002)”.

In Table 4, the reference for the row “Recombinant Vaccines 3” was incorrectly listed as “Ogg et al. (2013)”. The correct reference is “Maes et al. (2006)”.

In Table 4, the reference for the row “DNA Vaccines 1” was incorrectly listed as “Vial et al. (2013)”. The correct reference is “Hooper et al. (2013)”.

In Table 4, the reference for the row “DNA Vaccines 2” was incorrectly listed as “Antonen et al. (2013)”. The correct reference is “Hooper et al. (2001)”.

In Table 4, the reference for the row “DNA Vaccines 3” was incorrectly listed as “Laine et al. (2015)”. The correct reference is “Hooper et al. (2006)”.

In Table 4, the reference for the row “DNA Vaccines 4” was incorrectly listed as “Vial et al. (2013)”. The correct reference is “Hooper et al. (2013)”.

In Table 4, the reference for the row “DNA Vaccines 5” was incorrectly listed as “Fire et al. (1998)”. The correct reference is “Brocato et al. (2013)”.

In Table 4, the reference for the row “DNA Vaccines 6” was incorrectly listed as “Safronetz et al. (2013)”. The correct reference is “Jiang et al. (2017)”.

In Table 4, the reference for the row “Subunit Vaccines” was incorrectly listed as “Ye et al. (2019)”. The correct reference is “Maes et al. (2008)”. The corrected Table 4 and its caption “Describing evaluation of Hantavirus vaccines in various animal models and some vaccines currently undergoing clinical trials.” appear below.

**Table 4:** Describing evaluation of Hantavirus vaccines in various animal models and some vaccines currently undergoing clinical trials.

**Vaccine type**	**Antigen**	**Animal model**	**Immunogenicity evaluation**	**References**
Inactivated vaccine	Formalin inactivated HNTV	Humans	Humoral response Neutralizing antibodies	Khan et al. (2019)
Virus-like particles	HTNV-VLP with CD40L or GM-CSF	Mice	Cytotoxic response Neutralization antibody Cytolytic activity	Dong et al. (2019)
	M	DHFR-deficient CHO cells	Antigen-specific IFN-γ production Effective against HTNV Still in developing phases	Dong et al. (2019)
Virus-vector vaccines	Replication-competent VSV-vectored SNV or ANDV glycoproteins	Syrian Hamster	Cross-reactive IgG antibodies Neutralizing antibodies	Warner et al. (2019)
	Replication-competent VSV-vectored ANDV glycoproteins	Syrian Hamsters	Neutralizing antibodies	Prescott et al. (2014)
	Non-replicating Ad vector expressing N, Gn, Gc, or Gn/Gc	Syrian Hamsters	CD8+ cell response Neutralizing antibodies	Safronetz et al. (2009)
Recombinant vaccines	Yeast-expressed DOBV nucleoprotein	Mice	NP-specific IgG response Th1/Th2 response Cross-reactivity with HTNV and PUUV	Geldmacher et al. (2004)
	Nucleoproteins from ANDV, TOPV, DOBV or PUUV	Bank voles	Specific CD8+ cell production Cross-reactive response against PUUV	de Carvalho et al. (2002)
	Truncated recombinant PUUV nucleoprotein linked to bacterial membrane protein	Mice	CD8+ T-cell response NP IgG response	Maes et al. (2006)
DNA vaccines	HTNV/PUUV/SNV/ANDV M gene segment mix	Rabbits	Neutralizing antibodies	Hooper et al. (2013)
	HTNV M segment	Rhesus macaques	Neutralizing antibodies Cross-reactivity with SEOV and DOBV	Hooper et al. (2001)
	ANDV and HNTV M gene segments	Rhesus macaques	Neutralizing antibodies	Hooper et al. (2006)
	SNV M gene segment	Syrian hamsters	Neutralizing antibodies	Hooper et al. (2013)
	PUUV M gene segment	Syrian hamsters	Protection against lethal ANDV infection, without nAbs Neutralizing antibodies	Brocato et al. (2013)
	Gn glycoprotein	BALB/c mice	Effective against HTNV Still in developing phases	Jiang et al. (2017)
Subunit vaccines	NP (nucleocapsid protein)	*E. coli* mutant ICONE NMRI mice	Effective against PUUV In developing Phases	Maes et al. (2008)

The authors apologize for these errors and state that they do not change the scientific conclusions of the article in any way. The original article has been updated.
